# NrCAM secreted by endometrial stromal cells enhances the progestin sensitivity of endometrial cancer cells through epigenetic modulation of *PRB*

**DOI:** 10.1038/s41417-022-00467-0

**Published:** 2022-04-06

**Authors:** Yali Cheng, Liying Xie, Zhiying Xu, Mengxin Hao, Bingyi Yang, Weiwei Shan, Yiqin Wang, Qiaoying Lv, Xiaojun Chen

**Affiliations:** 1 Department of Gynecology, Obstetrics and Gynecology Hospital of Fudan University, Shanghai, China; 2 Shanghai Key Laboratory of Female Reproductive Endocrine Related Diseases, Fudan University, Shanghai, P. R. China; 3 Department of Pathology, Obstetrics and Gynecology Hospital of Fudan University, Shanghai, China

**Keywords:** Endometrial cancer, Drug delivery, Endometrial cancer

## Abstract

Progestin is one of the main hormone treatment regimens for early-stage estrogen receptor- and progesterone receptor (PR)-positive endometrial cancer (EC). However, the response rate of EC to progestins is unsatisfactory. Investigating the mechanisms related to progestin treatment could help improve treatment efficacy. Studies have demonstrated that normal endometrial stromal cells (ESCs) increase the inhibitory effect of progestin on EC cell proliferation via paracrine signaling, but the mechanisms involved remain unclear. In this study, we found that ESCs had different morphological features between progestin-sensitive and -insensitive EC tissues. ESCs presented typical decidualization changes in progestin-sensitive cases, while they remained slim in progestin-insensitive EC lesions, indicating no response. Furthermore, ESCs enhanced the inhibitory effect of medroxyprogesterone acetate (MPA) on EC cell proliferation by secreting neuron cell adhesion molecule (NrCAM). MPA treatment enhanced NrCAM secretion by ESCs. EC xenografts in BALB/C nude mice demonstrated that MPA combined with NrCAM had an increased tumor inhibitory effect compared with MPA or NrCAM alone. Mechanistically, MPA upregulated NrCAM expression in ESCs through PR. Specifically, NrCAM increased PR expression in EC cells through TET1-induced hydroxymethylation of the *PRB* gene promoter region. These findings indicate that NrCAM or NrCAM combined with progestins could be a new EC treatment.

## Introduction

Endometrial cancer (EC) is one of the most common malignant neoplasms of the female reproductive system, with an estimated 65,950 new cases in the USA in 2022 [1]. Prolonged estrogen exposure without progestin protection is the primary oncogenic mechanism of EC [2]. Progestins are the first-line therapy for early-stage EC patients receiving fertility-preserving treatment as well as an alternative choice for advanced or recurrent EC with positive estrogen receptor α (ERα) and progesterone receptor (PR) expression. For early-stage EC, the complete response rate for fertility-preserving treatment using high-dose progestins is 70–80% [3]. However, for advanced EC patients, the response rate is as low as 15–20% [4]. Therefore, it is crucial to understand the mechanisms of how progestin treatment impacts EC cells for improving treatment efficacy.

Previous studies focused on the direct effect of progestins on EC. It has been reported that progestins exert anticancer effects through several mechanisms, including antagonizing estrogen signaling, inhibiting the PI3K/AKT and WNT/β-catenin pathways [5, 6], upregulating the cell cycle-dependent inhibitory factors P21 and P27, and inhibiting expression of activator protein-1 and matrix metalloproteinases [7, 8]. In addition to these direct effects of progestins on EC cells, the endometrial stromal microenvironment might be involved in regulating progestin sensitivity in EC.

Despite being a key component of the endometrium, the role and mechanism of endometrial stromal cells (ESCs) in regulating progestin activity on endometrial epithelial cells has not been adequately studied. Previous studies have reported that ESCs improve the inhibitory effect of progestin on EC cells. Positive PR expression in ESCs is imperative for the inhibition of estrogen-induced DNA synthesis and endometrial epithelial cell proliferation in the presence of progestins [9, 10]. Paracrine factors derived from ESCs have been shown to inhibit estradiol-stimulated EC cell proliferation by regulating PI3K/AKT/survivin signaling or inactivating estrogen activity by increasing 17-β hydroxysteroid dehydrogenase enzymatic activity [11–13]. How ESCs regulated the response of EC cells to progestins is of great interest. Furthermore, identifying the paracrine factors secreted by ESCs that enhance the progestin sensitivity of EC could improve treatment efficacy.

In this study, we found that ESCs enhanced the inhibitory effect of medroxyprogesterone acetate (MPA) on EC cells by secreting neuron cell adhesion molecule (NrCAM). MPA upregulated NrCAM expression in ESCs through PR signaling. Furthermore, MPA combined with NrCAM had a better tumor inhibitory effect than MPA or NrCAM alone. Mechanistically, NrCAM upregulated PR expression via TET1-induced hydroxymethylation of the *PRB* gene promoter region in EC cells, which sensitized EC cells to progestin treatment. Our findings indicate that NrCAM could be a new EC treatment.

## Materials and methods

### Clinical case collection

Representative cases of progestin-sensitive (*n* = 5) and progestin-insensitive (*n* = 4) EC patients who received oral megestrol acetate (MA) (160 mg/day) and/or levonorgestrel intrauterine system (LNG-IUS) fertility-preserving treatment between June 2017 and December 2020 were used for pathological examination. The basic characteristics of patients were described in Supplementary Table 1. The definitions of “progestin-sensitive” and “progestin-insensitive” are referred to our previous study [14]. Briefly, “progestin-insensitive” was defined as meeting one of the following criteria: (1) presented with progressive disease at any time during conservative treatment; (2) stable disease after 7 months of treatment; and/or (3) did not achieve complete response after 10 months of treatment. All patients were pathologically diagnosed using endometrial biopsy through dilation and curettage with or without hysteroscopy. The inclusion criteria for conservative treatment follow the National Comprehensive Cancer Network guidelines [15]. Pathological diagnosis was confirmed by two experienced gynecological pathologists according to the World Health Organization pathological classification (2020) [16].

### Endometrial tissue collection and isolation of ESCs

ESCs were extracted from normal endometrium samples obtained from 30 premenopausal patients undergoing total hysterectomy due to leiomyoma, cervical high-grade squamous intraepithelial lesion, or cervical cancer at the Obstetrics and Gynecology Hospital of Fudan University between January 2016 and December 2020. No patients had received hormone therapy for 6 months before surgery. Pathological diagnosis was independently confirmed by at least two pathologists. Patient details are shown in Supplementary Table 2.

Endometrial samples were minced into small pieces (<1 mm in diameter), subjected to mild collagenase digestion, and ESCs were isolated as previously described [17]. Then ESCs were identified as Vimentin-positive and Keratin-negative. Whether ESCs were acquired at the proliferative phase or secretory phase was determined according to the date of the menstrual cycle when the patient received operation and the postoperative pathological diagnostic report. Totally 18 ESCs were acquired at proliferative phase and 12 at secretory phase. There was no significant difference in the basic information of the patients (age, BMI, and pathological diagnosis) between the proliferative phase and secretory phase (Supplementary Tables 2, 3).

### Cell lines, cell culture, and preparation of conditioned medium (CM)

The human EC cell line Ishikawa was kindly provided by Dr. Yu Yinhua (MD Anderson Cancer Center, Houston, TX, USA), the human EC cell line ECC-1 and human fetal lung fibroblast 1 (HLF-1) were purchased from American Type Culture Collection (Manassas, VA, USA). Ishikawa and ECC-1 cells were authenticated by short-tandem-repeat (STR) profiling (DNA fingerprinting) in December 2019. Ishikawa and HLF-1 were cultured in DMEM/F12 (HyClone, Logan, UT, USA) supplemented with 10% fetal bovine serum (FBS; Gibco, Waltham, MA, USA) and 1% Antibiotic–Antimycotic. ECC-1 cells were cultured in RPMI 1640 medium (Hyclone) supplemented with 10% FBS and 1% Antibiotic–Antimycotic. Prior to MPA intervention, cells were cultured in phenol red-free medium supplemented with 2% charcoal-stripped FBS (Biological Industries, Kibbutz Beit-Haemek, Israel). CM was collected from the indicated cells after 48 h of culture, then centrifuged at 1000 rpm to remove cellular debris, and stored at −80 °C.

### Drug treatment

Drugs used in this study included MPA (Sigma-Aldrich, St. Louis, MO, USA), recombinant human NrCAM protein (CC04, NovoProtein, Shanghai, China), recombinant human BMP2 protein (C012, NovoProtein), recombinant human WISP1 protein (10442-H08H, Sino Biological, Beijing, China), and recombinant human ITGA10 protein (5895-AB-050, R&D Systems, Minneapolis, MN, USA) at the indicated doses for the indicated periods. Vehicles used included ethanol (EtOH) and distilled water.

### Immunocytochemical (ICC) staining

ICC staining was performed as previously described [18]. The primary antibodies included anti-Vimentin (1:200; #5741, Cell Signaling Technology, Danvers, MA, USA) and anti-Keratin antibody (1:200; #4545, Cell Signaling Technology).

### siRNA transfection

The siRNAs of si*PGR*, si*NrCAM*, and si*TET1* were designed and constructed by RiboBio, (Guangzhou, China). Target sequences used for gene silencing are listed in Supplementary Table 4. The siRNAs were transfected using Lipofectamine-3000 (Invitrogen, Waltham, MA, USA). The siCtrl sequence was used as a transfection control. The silencing efficiency was verified by real-time PCR and western blotting.

### Western blotting

The western blotting protocol was as previously described [19]. The primary antibodies included anti-PGR (1:200; SC-166169, Santa Cruz Biotechnology, Dallas, TX, USA), anti-NrCAM (1:1000; 21608-1-AP, Proteintech, Rosemont, IL, USA), and anti-GAPDH (#5174, Cell Signaling Technology, USA).

### Enzyme-linked immunosorbent assay (ELISA)

NrCAM concentrations in CM derived from ESCs were quantified using a human NrCAM ELISA kit (CUSABIO, Wuhan, China). This kit has a validated detection range of 78–5000 pg/mL. The intra- and inter-assay coefficients of variation for all assays were <8% and 10%, respectively.

### Real-time PCR and RNA sequencing (RNA-Seq)

RNA extraction and real-time PCR were conducted as previously described [20]. The primers were designed and synthesized by Sangon Biotech (Shanghai, China), and primer sequences were listed in Supplementary Table 5. RNA-Seq was performed by Genergy Bio-Technology (Shanghai, China).

### Dot blot and hydroxymethylated DNA immunoprecipitation (hMeDIP) assays

Dot blot and hMeDIP assays were used to assess hydroxymethylation levels of total DNAs and the *PRB* promoter, respectively. Total DNAs were extracted using the QIAamp DNA Mini Kit (QIAGEN, Hilden, Germany). Dot blot and hMeDIP assays were performed as previously described [21, 22]. Briefly, 1 μL of DNAs (500 ng) was dropped on a nitrocellulose filter membrane and irradiated with a UV lamp (2000 W) for 15 min to cross-link DNAs, and incubated with 5-hydroxymethylcytosine (5-hmC) primary antibody overnight and then with secondary antibody (Jackson ImmunoResearch, West Grove, PA, USA), and detected with ECL regent. Methylene blue (MB) was stained as a loading control. The 5-hmC antibody was purchased from Active Motif (55010, Carlsbad, CA, USA), rabbit IgG was used as a negative control. Isolated DNA fragments were evaluated by real-time PCR with the *PRB* promoter fragment primers (Supplementary Table 6).

### Cell counting Kit-8 (CCK-8) assay and EdU staining

The CCK-8 (Dojindo, Kumamoto, Japan) and 5-Ethnyl-2′-deoxyuridine (Cell-Light EdU Cell Proliferation Detection Kit (RiboBio) assays were used to evaluate cell viability and proliferation [23].

### Establishment of uterine horn xenograft tumors

Female athymic BALB/C nude mice weighing 18–22 g (*n* = 24) were purchased from the Laboratory Animal Science Department of Shanghai Medical College, Fudan University (Shanghai, China). Mice were maintained in a specific pathogen-free and temperature-controlled facility on a 12 h light/dark cycle. All procedures were performed as previously described [24]. All mice were anesthetized with 200 μL nembutal (i.p.), and a 1-cm incision was made on the left and right lower flanks to expose the uterine horn in mice. A single-cell suspension of Ishikawa cells (5 × 10^5^ cells) was injected into each of the left and right uterine horn lumen, and the incisions were closed with staples. After 2 weeks, mice were randomly divided into the following four groups: control (Ctrl), MPA, NrCAM, and MPA + NrCAM (*n* = 6 per group). Mice were treated with 5 mg/kg NrCAM (CC04, NovoProtein) in PBS (i.p.) every 2 day and/or 100 mg/kg MPA (Sigma-Aldrich) in corn oil by gavage every day at a dose of 0.1 mL/10 g (body weight). The vehicles used in the Ctrl group were PBS (i.p.) and corn oil (gavage). Animals were euthanized after NrCAM and/or MPA treatment for 16 days, and the weight of bilateral uterine horns including tumors was recorded. Tumor tissue was fixed in formalin for histological analysis.

### Hematoxylin and eosin (H&E) staining and immunohistochemical (IHC) staining

H&E staining and IHC staining were performed as previously described [25] with the following primary antibodies: anti-Ki67 (ab16667, Abcam, Cambridge, UK), anti-PR (ab2765, Abcam), and anti-5-hmC (GT13612, GeneTex, Irvine, CA, USA). Semi-quantitative optical analysis was performed as previously described [22].

### Statistical analysis

Statistical analyses were performed with SPSS v22.0 (IBM, Armonk, NY, USA). Volcano plots were constructed using R software, v3.5.2 (R Foundation, http://www.r-project.org/) to screen out differentially expressed genes. Student’s *t* test and ANOVA were used for further analyses. All statistical tests were two-sided, and *P* values <0.05 were considered statistically significant. Each experiment was repeated at least three times. Error bars in the graphs indicate standard deviation.

## Results

### ESCs enhanced the inhibitory effect of progestins on EC cell proliferation

**Fig. 1:**
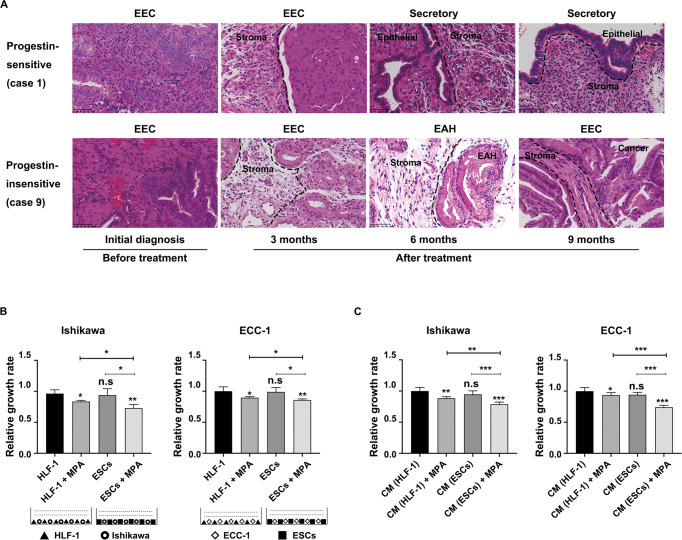
ESCs enhanced the inhibitory effect of progestins on EC cell proliferation. **A** Representative pathological images of EC before treatment, and 3, 6, and 9 months after progestin therapy. H&E staining of progestin-sensitive EC specimens (top panel) and progestin-insensitive EC specimens (bottom panel) was performed before and after progestin therapy. Progestin-sensitive EC case 1 was initially diagnosed as EEC before progestin therapy, then diagnosed as EAH (3 months after treatment) and secretory endometrium (6 and 9 months after treatment). Progestin-insensitive EC case 9 was initially diagnosed as EEC before progestin therapy, then diagnosed as EEC (3 months after treatment), EAH (6 months after treatment), and EEC (9 months after treatment). Stromal cells showed typical decidualization change (stromal cell edema) in progestin-sensitive cases, while they remained slim in progestin-insensitive cases. Original magnification, ×400; Scale bar, 50 μm. **B**, **C** ESCs enhanced the inhibitory effect of MPA on EC cell proliferation through both direct contact and their CM. Ishikawa and ECC-1 cells were cocultured with ESCs or HLF-1 with or without 10 μM MPA for 48 h before CCK-8 assays (**B**). Ishikawa and ECC-1 cells were cultured with CM of ESCs/HLF-1 with or without 10 μM MPA for 48 h before CCK-8 assays (**C**). EEC endometrioid endometrial cancer, EAH endometrial atypical hyperplasia, ESCs endometrial stromal cells, HLF-1 human fetal lung fibroblast 1, MPA medroxyprogesterone acetate, CM conditioned medium. *P < 0.05; **P < 0.01; ***P < 0.001; n.s. not significant.

To explore the role of ESCs in progestin-based EC treatment, we first investigated the pathological changes of ESCs in EC patients who received progestin treatment (MA and/or LNG-IUS). In total, nine patients with endometrium-confined grade 1 endometrioid EC (EEC) were analyzed. Basic characteristics of patients are described in Supplementary Table 1. We found that ESCs had different morphological features between progestin-sensitive and progestin-insensitive cases. ESCs presented typical decidualization changes (stromal cell edema) in progestin-sensitive cases, while they remained slim in progestin-insensitive EC lesions, indicating no response (Fig. 1A). These findings indicated that ESCs might regulate the response to progestin treatment.

To determine whether ESCs increased the inhibitory effect of progestins on EC cell proliferation, Ishikawa and ECC-1 cells were cocultured with ESCs, HLF-1 (negative control), or CM from ESCs or HLF-1 (Fig. 1B, C). ESCs were isolated from normal endometrium and then identified as Vimentin-positive and Keratin-negative by ICC staining (Supplementary Fig. 1A). Then HLF-1 was used as negative control and the proliferation rates of ESCs and HLF-1 were similar at 48 h culture (Supplementary Fig. 1B), so we considered that the differences in proliferation rates in the coculture system were due to EC cell proliferation. Our results showed that either direct coculture or CM from ESCs both inhibited EC cell proliferation. Interestingly, EC cell proliferation was most significantly inhibited in coculture system with ESCs in the presence of MPA (Fig. 1B). Furthermore, CM from ESCs also enhanced the inhibitory effect of MPA on EC cell proliferation (Fig. 1C). This suggested that paracrine factors from ESCs enhanced progestin response.

In order to determine whether ESCs derived from the proliferative phase and secretory phase had similar effects on EC cell growth, the function of ESCs from case 1 to case 11 (7 cases in the proliferative phase and 4 cases in secretory phase) were verified by CCK-8 test in EC cells. The results showed that the inhibitory effect of ESCs on EC cell growth was consistent as shown in Supplementary Fig. 2. We thus confirmed that the ESCs derived from either proliferative or secretory endometrium had consistent inhibitory effects on EC cells.

### Progestin-PR signaling induced ESCs to secrete paracrine factors that inhibited EC cell proliferation

**Fig. 2:**
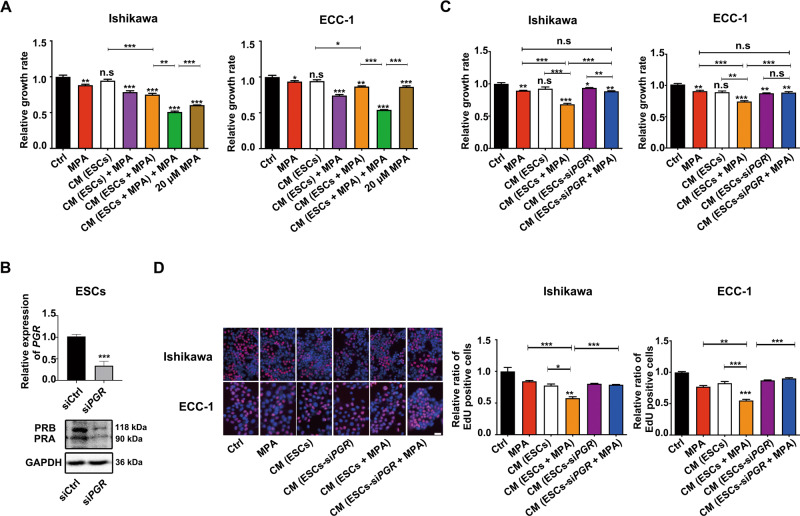
Progestin-PR signaling-mediated secretion of paracrine factors by ESCs inhibited EC cell proliferation. **A** CM derived from MPA-pretreated ESCs and MPA cotreatment showed the greatest inhibitory effect on EC cell growth compared with 20 μM MPA group and CM (ESCs) + MPA group. CM was extracted from ESCs treated with or without 10 μM MPA for 48 h. Ishikawa and ECC-1 cells were cultured with CM and/or 10 μM MPA for 48 h before CCK-8 assays. **B** ESCs were transfected with siRNAs targeting *PGR*. Real-time PCR and western blotting were used to evaluate *PGR* mRNA and PR protein (PRA and PRB) expression. **C**, **D** The inhibitory effect of CM (ESCs-si*PGR* + MPA) on EC cell proliferation was compromised compared with CM (ESCs + MPA) group. Ishikawa and ECC-1 cells were cultured with the indicated CM for 48 h before CCK-8 and EdU assays. Scale bar, 300 μm. *P < 0.05; **P < 0.01; ***P < 0.001; n.s. not significant.

We then determined whether progestins promoted ESCs to secrete paracrine factors that inhibited EC cell proliferation. ESCs were pretreated with 10 μM MPA for 48 h, and CM was then collected and used to treat Ishikawa and ECC-1 cells. CCK-8 results showed that CM derived from MPA-pretreated ESCs inhibited EC cell proliferation more significantly than CM from untreated ESCs (Fig. 2A). Moreover, CM derived from MPA-pretreated ESCs supplemented with MPA had the highest growth inhibitory rate (Ishikawa, 49.12%, *P* < 0.001; ECC-1, 45.94%, *P* < 0.001). To confirm that paracrine factors from MPA-pretreated ESCs (rather than increased MPA concentrations) were responsible for the enhanced inhibition of EC cell proliferation, we compared the inhibitory effects of CM derived from 10 μM MPA-pretreated ESCs and 10 μM MPA cotreatment or 20 μM MPA alone on EC cell growth. The cotreatment group showed greater inhibitory effect than 20 μM MPA alone (Ishikawa: 49.12% vs. 39.53%, *P* < 0.001; ECC-1: 45.94% vs. 14.61%, *P* < 0.001) (Fig. 2A). These findings indicated that paracrine factors from progestin-pretreated ESCs significantly inhibited EC cell proliferation.

We then investigated whether progestins promoted the secretion of paracrine factors through PR in ESCs. Then PR was silenced in ESCs by transfecting siRNAs targeting *PGR*. Real-time PCR, and western blotting of PR protein (PRA and PRB) were used to verify the interference effect (Fig. 2B). CCK-8 and EdU assays showed that the inhibitory effect of CM from MPA-pretreated ESCs on EC cells proliferation was compromised after silencing *PGR* in ESCs (Fig. 2C, D). This indicated that progestin-PR signaling in ESCs mediated secretion of the paracrine factors that inhibited EC cell proliferation.

### Activating progestin-PR signaling in ESCs increased PR expression in EC cells via hydroxymethylation of the *PRB* promoter

**Fig. 3:**
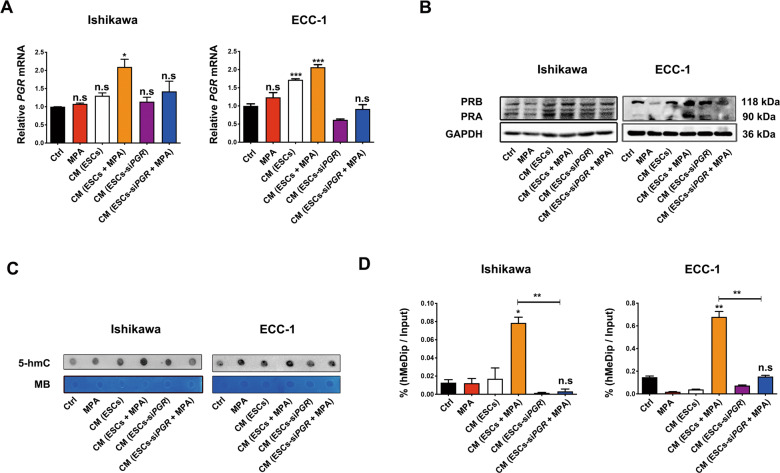
Progestin-PR signaling in ESCs increased PR expression in EC cells via hydroxymethylation of the PRB promoter. **A**, **B** CM (ESCs-si*PGR* + MPA) attenuated the upregulation of CM (ESCs + MPA) on *PGR* mRNA and PR protein expression in EC cells. CM was extracted from ESCs and ESCs-*siPGR* with or without 10 μM MPA treatment for 48 h. Then Ishikawa and ECC-1 cells were treated with the indicated CM for 24 or 48 h, respectively. *PGR* mRNA and PR protein expression were analyzed by real-time PCR and western blotting, respectively. GAPDH was used as the normal control. **C** CM (ESCs-si*PGR* + MPA) attenuated the upregulation of CM (ESCs + MPA) on genomic DNA hydroxymethylation. Then Ishikawa and ECC-1 cells were cultured with the indicated CM for 24 h. 5-hmC levels of total genomic DNAs were assessed by dot blot analysis. **D** CM (ESCs-si*PGR* + MPA) attenuated the upregulation of CM (ESCs + MPA) on hydroxymethylation levels of the *PGR* promoter in EC cells. After 24 h of treatment with the indicated CM, Ishikawa and ECC-1 cells were harvested to assess 5-hmC levels in the *PRB* promoter fragment. *P < 0.05; **P < 0.01; ***P < 0.001; n.s. not significant.

As PR status in EC cells is vital for progestin response, we asked whether CM from progestin-pretreated ESCs increased progestin sensitivity of EC cells via upregulating PR expression. EC cells were cultured with CM derived from ESCs or ESCs transfected with si*PGR* with or without MPA pretreatment. We found that CM from MPA-pretreated ESCs significantly upregulated *PGR* mRNA and PR protein expression in EC cells, whereas this effect was diminished when PR expression was silenced in ESCs (Fig. 3A, B). This suggested that CM from MPA-pretreated ESCs might increase progestin sensitivity of EC cells via upregulating PR. We thus focused on potential mechanisms through which CM from ESCs upregulated PR expression in EC cells.

Hydroxymethylation is an important mechanism for regulating PR expression in EC cells. We examined whether CM from MPA-pretreated ESCs increased PR expression in EC cells through hydroxymethylation of *PGR*. We cultured EC cells with CM from ESCs or ESCs-si*PGR* with or without 10 μM MPA pretreatment. After 48 h, 5-hmC levels of genomic DNAs were analyzed in EC cells by dot blot. The results showed that CM from MPA-pretreated ESCs significantly increased hydroxymethylation of genomic DNAs in EC cells, which were reduced after silencing *PGR* in ESCs (Fig. 3C). As PRB is main PR isoform responsible for progestin-mediated inhibitory effects on EC cells, we examined the hydroxymethylation level of *PRB* promoter by hMeDIP assay. Similarly, CM from MPA-pretreated ESCs significantly increased hydroxymethylation levels of *PRB* promoter in EC cells, but this effect diminished after silencing *PGR* in ESCs. These findings indicated that progestin-PR signaling in ESCs increased PR expression in EC cells through *PRB* hydroxymethylation.

### NrCAM secreted by progestin-pretreated ESCs mediated the inhibitory effect of ESCs on EC cell proliferation

**Fig. 4:**
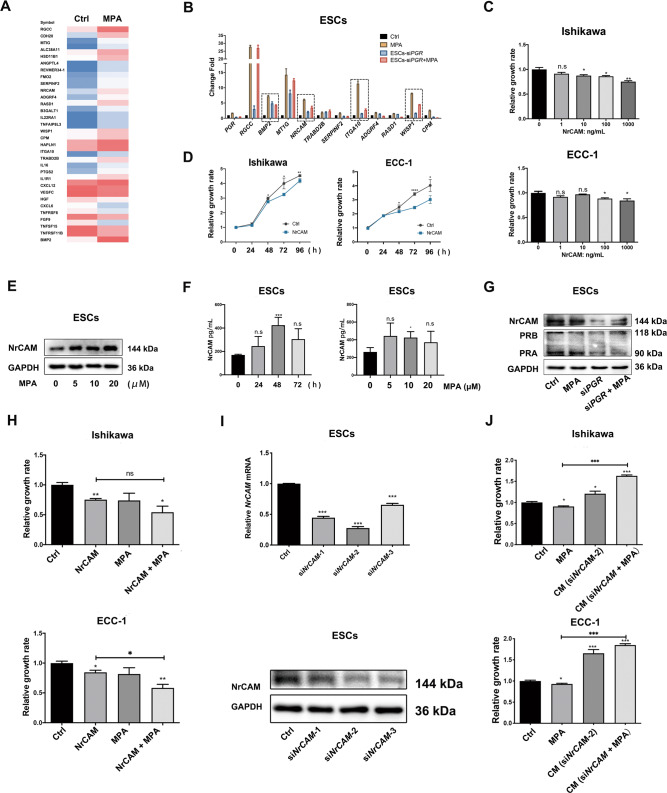
NrCAM from progestin-pretreated ESCs mediated the inhibitory effect of ESCs on EC cell proliferation. **A** Cluster analysis of MPA-regulated genes encoding secretory proteins in ESCs. ESCs were treated with 10 μM MPA or ethanol (EtOH) for 6 h before RNA sequencing. **B** mRNA levels of *NrCAM*, *BMP2*, *WISP1*, and *ITGA10* were significantly upregulated in ESCs after MPA treatment. Silencing PR expression with si*PGR* in ESCs weakened MPA-induced upregulation of these four proteins. ESCs or ESCs-si*PGR* were treated with or without 10 μM MPA for 6 h. Eleven candidate gene mRNA levels were reevaluated by real-time PCR. **C** Exogenous NrCAM inhibited EC cell proliferation in a dose-dependent manner. Ishikawa and ECC-1 cells were treated with 0, 1, 10, 100, and 1000 ng/mL NrCAM for 48 h before CCK-8 assays. **D** Exogenous NrCAM inhibited EC cell proliferation in a time-dependent manner. Ishikawa and ECC-1 cells were treated with 1000 ng/mL NrCAM for 24, 48, and 72 h before CCK-8 assays. **E** MPA promoted NrCAM protein expression in ESCs in a dose-dependent manner. NrCAM expression was detected by western blotting. ESCs were treated with 0, 5, 10, and 20 μM MPA for 48 h. **F** MPA promoted NrCAM secretion in ESCs by ELISA. ESCs were treated with MPA at the indicated dose for 48 h (left) or 10 μM MPA for 24, 48, or 72 h (right). The CM extracted from ESCs was collected to measure NrCAM concentration by ELISA. **G** MPA-induced NrCAM protein expression was attenuated by silencing *PGR* in ESCs. ESCs or ESCs-si*PGR* were treated with 10 μM MPA for 48 h before western blotting analysis. **H** NrCAM and MPA cotreatment had a stronger inhibitory effect on EC cell proliferation than MPA or NrCAM alone. Ishikawa and ECC-1 cells were treated with 1000 ng/mL NrCAM and/or 10 μM MPA for 48 h before CCK-8 assays. **I** Transfection efficiency of siRNAs targeting *NrCAM* was confirmed by real-time PCR and western blotting. **J** The inhibitory effect of ESCs on EC cell proliferation was blocked by silencing NrCAM expression in ESCs. After transfection with si*NrCAM* or siCtrl for 8 h, ESCs were treated with or without 10 μM MPA for 48 h, then for CM collection. Ishikawa and ECC-1 cells were treated with 10 μM MPA, CM (ESCs-si*NrCAM*-2) and CM (ESCs-si*NrCAM*-2 + MPA) for 48 h before CCK-8 assays. *P < 0.05; **P < 0.01; ***P < 0.001; n.s. not significant.

To investigate secretory factors from MPA-pretreated ESCs that mediated the inhibitory effect of ESCs on EC cells proliferation, ESCs before and after 10 μM MPA treatment were analyzed by RNA-Seq. The top 50 differentially expressed genes with |log2 Fold Change | ≥1 and *P* < 0.05 are shown in Fig. 4A. Eleven secretory proteins were possible candidates including RGCC, BMP2, MT1G, NrCAM, TRABD2B, SERPINF2, ITGA10, ADGRF4, RASD1, WISP1, and CPM using Signal IP 3.0 software (Fig. 4B). Real-time PCR confirmed that NrCAM, BMP2, WISP1, and ITGA10 mRNA levels were significantly increased in MPA-treated ESCs. The MPA-induced upregulation of these four proteins was attenuated when PR was silenced by si*PGR* in ESCs (Fig. 4B). Therefore, we speculated that NrCAM, BMP2, WISP1, and ITGA10 were potential candidate factors secreted by ESCs to inhibit EC cell proliferation.

First, we analyzed the proliferation rates of EC cells following individual treatment with the four secretory factors. Only exogenous NrCAM significantly inhibited the proliferation of EC cells in a dose- and time-dependent manner (Fig. 4C, D and Supplementary Fig. 3A). In addition, MPA increased NrCAM protein expression in ESCs (Fig. 4E). ELISA results of CM from ESCs also confirmed that NrCAM secretion was increased after MPA treatment (Fig. 4F). MPA-induced NrCAM protein expression was attenuated by silencing *PGR* in ESCs (Fig. 4G). moreover, cotreatment with exogenous NrCAM (1000 ng/mL) and MPA (10 μM) exhibited a stronger inhibitory effect on EC cell proliferation than MPA or NrCAM alone (Fig. 4H). However, BMP2 and ITGA10 compromised the inhibitory effect of MPA on EC cell proliferation, while WISP1 did not have an effect (Supplementary Fig. 3B). Furthermore, the inhibitory effect of ESCs on EC cell proliferation was blocked by transfecting siRNAs targeting *NrCAM* in ESCs (Fig. 4I, J). This suggested that NrCAM was the secretory protein from ESCs responsible for the increased sensitivity of EC cells to progestins.

### NrCAM increased PR expression in EC cells via TET1-mediated hydroxymethylation of the *PRB* promoter

**Fig. 5:**
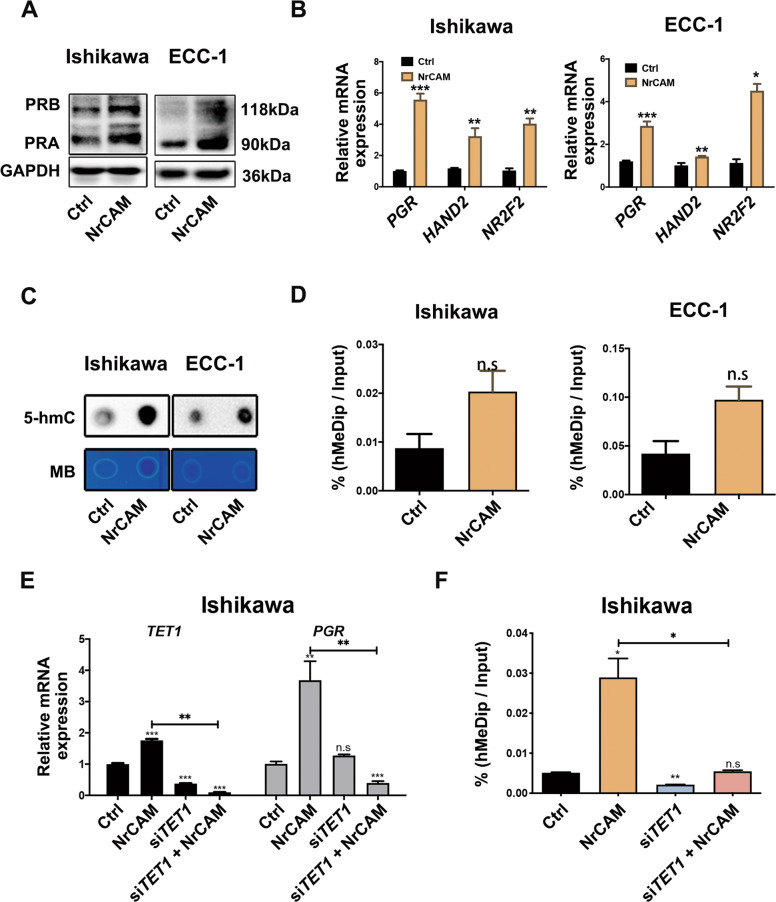
NrCAM increased PR expression in EC cells via TET1-mediated hydroxymethylation of the PRB promoter. **A** Exogenous NrCAM promoted PR protein expression in EC cells. Ishikawa and ECC-1 cells were treated with 1000 ng/mL NrCAM for 48 h. **B** Exogenous NrCAM increased transcription of *PGR* and its downstream target genes *HAND2* and *HR2F2* in EC cells. After treatment with 1000 ng/mL NrCAM for 12 h, Ishikawa and ECC-1 cells were analyzed by real-time PCR. **C** NrCAM increased genomic DNA hydroxymethylation in EC cells. Ishikawa and ECC-1 cells were treated with 1000 ng/mL NrCAM for 24 h, and then 5-hmC levels of total DNAs were assessed by dot blot. **D** NrCAM promoted hydroxymethylation levels of the *PRB* promoter in EC cells. After treatment with 1000 ng/mL NrCAM for 24 h, 5-hmC levels of the *PGR* promoter fragment from Ishikawa and ECC-1 cells were detected by hMeDIP. **E** NrCAM increased *TET1* and *PGR* mRNA levels in EC cells. Silencing TET1 reduced NrCAM-induced PR expression in EC cells. After transfecting si*TET1*, Ishikawa cells were treated with 1000 ng/mL NrCAM for 24 h, then were harvested for real-time PCR analysis. **F** Silencing *TET1* blocked NrCAM-induced hydroxymethylation levels of the *PRB* promoter in EC cells. After transfecting with siRNAs targeting *TET1*, Ishikawa cells were treated with 1000 ng/mL NrCAM for 24 h. Then 5-hmC levels of the *PGR* promoter fragment were detected by hMeDIP. *P < 0.05; **P < 0.01; ***P < 0.001; n.s. not significant.

As shown above, CM from progestin-pretreated ESCs increased PR expression in EC cells through hydroxymethylation of *PRB*. Whether NrCAM mediated the regulation of *PRB* hydroxymethylation in EC cells were further analyzed. EC cells were cultured with exogenous NrCAM protein (1000 ng/mL) for western blotting and Real-time PCR, respectively. Results showed that NrCAM promoted *PGR* mRNA and PR protein expression in EC cells (Fig. 5A, B). HAND2 and NR2F2, which are downstream targets of PR signaling in EC cells, were also upregulated after NrCAM treatment (Fig. 5B). These results suggested that NrCAM increased PR expression and stimulated PR signaling in EC cells.

We then asked whether NrCAM inhibited EC cell proliferation via hydroxymethylation of *PRB*. EC cells were treated with NrCAM (1000 ng/mL) for 24 h before dot blot and hMeDIP assays. Dot blot results showed a significant increase in genomic DNA hydroxymethylation levels in EC cells (Fig. 5C), and hMeDIP results demonstrated increased levels of hydroxymethylation in the *PRB* promoter region in EC cells (Fig. 5D). TET1 is one of the primary components of the ten-eleven translocation 5-methylcytosine (5-mc) dioxygenase family and catalyzes the conversion of 5-mC to 5-hmC [26]. NrCAM increased TET1 mRNA levels in EC cells, and silencing TET1 expression diminished the upregulated effect of NrCAM on PR expression in EC cells (Fig. 5E). Similarly, silencing TET1 expression in EC cells compromised the ability of NrCAM to induce *PRB* hydroxymethylation (Fig. 5F). Thus, NrCAM increased PR expression through TET1-induced hydroxymethylation of the *PRB* promoter in EC cells.

### NrCAM inhibited EC tumor growth in a murine xenograft model

**Fig. 6:**
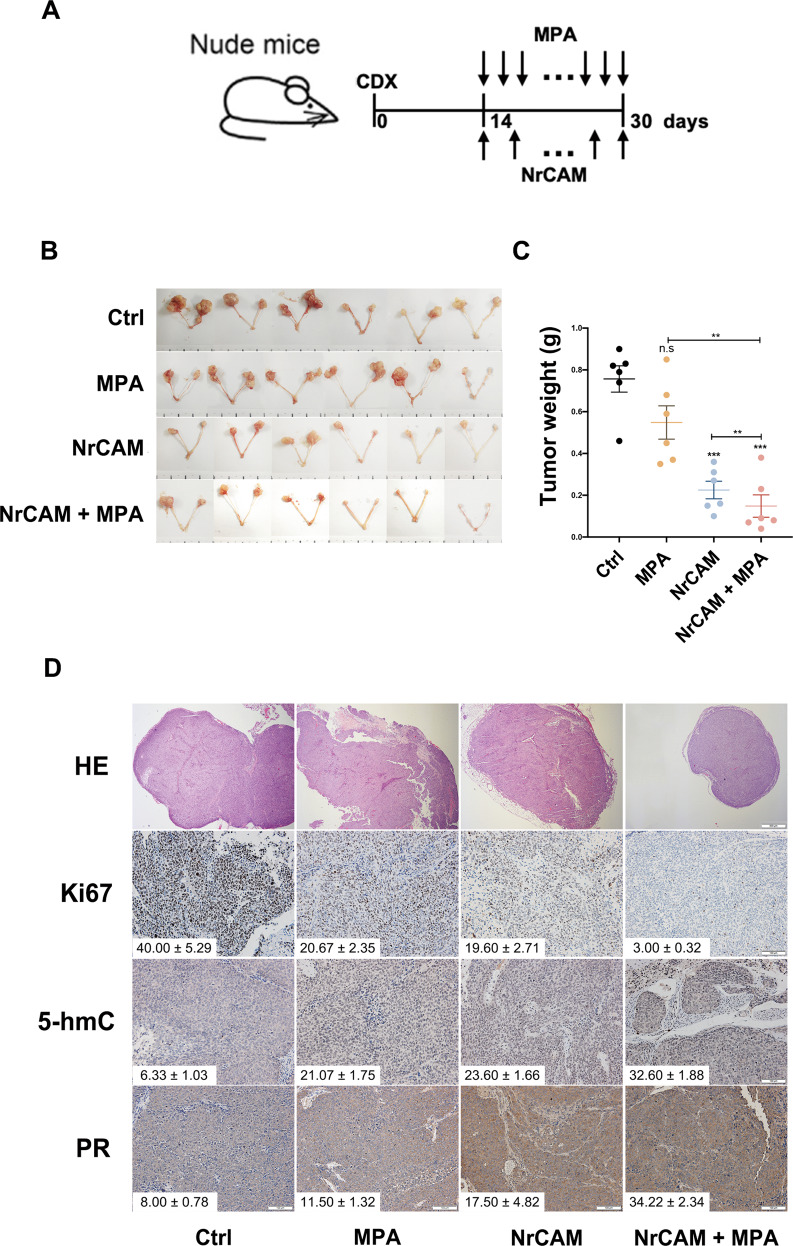
NrCAM inhibited EC xenograft growth. **A** A single-cell suspension of 5 × 10^5^ cells was injected into the uterine horns of nude mice. After 14 days, the mice were treated with 5 mg/kg NrCAM in PBS (i.p.) every 2 d and/or 100 mg/kg MPA in corn oil by gavage at a dose of 0.1 mL/10 g daily for another 16 days, and then were sacrificed. **B**, **C** NrCAM+MPA had the highest inhibitory effect on EC uterine horn xenograft growth compared with NrCAM or MPA alone. Images of uterine horn xenografts (**B**). Weight of uterine horn xenografts in different groups (**C**). Ruler unit, 1 mm. **D** IHC staining for Ki67, PR, and 5-hmC in EC xenograft tumor tissues. The IHC staining score was calculated by semi-quantitative optical analysis. Ki67 score (*n* = 5 each group; NrCAM + MPA versus MPA, *P* < 0.001; NrCAM + MPA versus NrCAM, *P* < 0.001); 5-hmC score (*n* = 5 each group; NrCAM+MPA versus MPA, *P* < 0.001; NrCAM +MPA versus NrCAM, *P* < 0.001); PR score (*n* = 5 each group; NrCAM+MPA versus MPA, *P* < 0.001; NrCAM +MPA versus NrCAM, P < 0.01). Original magnification, ×200. CDX, cell-derived xenograft. *P < 0.05; **P < 0.01; ***P < 0.001; n.s. not significant.

Finally, we evaluated the effect of NrCAM with or without MPA on the growth of uterine horn EC xenografts in BALB/C nude mice. Tumor size and weight of bilateral uterine horns including tumors were evaluated after 16-day NrCAM and/or MPA treatment (Fig. 6A). The results showed that both NrCAM and NrCAM + MPA groups inhibited EC xenograft growth, and NrCAM + MPA group had the highest inhibitory efficiency compared with NrCAM or MPA alone (Fig. 6B, C). IHC staining showed lower Ki67 levels and increased levels of PR and 5-hmC in NrCAM and NrCAM + MPA groups compared with MPA and Ctrl groups (Fig. 6D). These findings indicated that NrCAM could be a promising therapy to improve progestin sensitivity in EC.

## Discussion

In this study, we demonstrated that ESCs play important roles in enhancing progestins’ ability to inhibit EC cell proliferation. NrCAM is the key protein secreted by ESCs in the presence of progestins that inhibits EC cell proliferation. Mechanistically, NrCAM upregulates PR expression in EC cells through TET1-induced hydroxymethylation of the *PRB* promoter region. Our findings indicated that NrCAM could be a new EC therapy.

Endometrial epithelial cells and ESCs are the two main components of the endometrium, but the roles and mechanisms of ESCs in maintaining endometrial homeostasis remain unclear [27, 28]. In addition to providing physical support, ESCs also regulate endometrial epithelial cell proliferation [29]. During menstrual cycles, ESCs suppresses the production of WNT ligands (Wnt4, Wnt5a, Frizzled2), insulin-like growth factor (IGF-1) and fibroblast growth factor, which regulate WNT and PI3K/AKT signaling in epithelial cells, thereby endometrial epithelial cell growth is inhibited [5, 30, 31]. Our results showed that ESCs enhanced the inhibitory effect of progestins on EC cell proliferation, indicating that ESCs play important roles not only mechanical support in both physiological and pathological circumstances. As the pathological grade of EC increases, the number of ESCs decreases gradually, which is the potential reason why progestins are insensitive in some EC cases with positive expression of ERα and PR in EC tissues.

Our study supports previous findings that positive PR expression in ESCs is conducive to their normal function. Using tissue recombinants prepared with uterine stroma and epithelium from wild-type and *PGR*-knockout mice, Kurita et al. demonstrated that the inhibitory effect of progestins on uterine epithelial DNA synthesis is mediated by stromal PR [10]. Janzen et al. found that signaling through stromal PR is necessary and sufficient for the antitumor effects of progestins on EC cells [9]. Our results also showed that the inhibitory effect of progestins on EC cell proliferation was compromised when *PGR* was silenced in ESCs.

Our study demonstrated that NrCAM is the key secretory factor mediating the inhibitory effect of ESCs on EC cell growth. Although studies have found that ESCs regulate EC in a paracrine manner [11, 32], our study is the first to report that NrCAM is the key secretory factor inhibiting EC cell growth and improving progestin sensitivity. NrCAM is a neuronal adhesion molecule with multiple immunoglobulin-like C2 domains and a fibronectin type III domain. This ankyrin-binding protein plays a broad role in neurodevelopment, including neuron-neuron adhesion, proliferation, differentiation, axon growth, signal transduction, synapse formation, and formation of myelinated nerve structures. NrCAM is also expressed in non-neural tissues, where it plays universal roles in cell–cell communication by transmitting signals from the intracellular domain to the actin cytoskeleton during cell-directed migration. The role of NrCAM varies in different cancers. It has been reported that NrCAM plays oncogenic effects in the development, migration, and invasion of the colon, pancreas, brain cancer, and melanoma [33–38]. Conversely, increased expression of NrCAM or CHL1 (a member of a homologous family) is correlated with better prognosis in prostate and breast cancer, both of which are hormone-related malignancies [39, 40]. Herein, we demonstrated that NrCAM secreted from ESCs effectively inhibited hormone-related EC proliferation. This study sheds light on the molecular mechanism of the inhibitory effect of ESCs on EC cell proliferation and may facilitate identifying novel therapeutic strategies for progestin-insensitive EC patients.

Our study demonstrated that NrCAM sensitized EC to progestins by upregulating PR expression via TET1-mediated hydroxymethylation of *PRB* in EC cells. It has been shown that CpG islands are enriched in the *PGR* promoter region and first exon; methylation modification at these sites plays a major role in suppressing PR expression [41, 42]. In 87% of EC specimens, *PGR* promoter hypermethylation was observed and was correlated with loss of PR protein expression [43]. Ghabreau et al. reported that PR expression was correlated with *PGR* promoter methylation in normal endometrium, hyperplasia and cancer [44], suggesting that epigenetic modification of *PGR* is involved in regulating progestin sensitivity in EC.

Our study had several limitations. Notably, we focused on ESCs derived from normal endometrium rather than EC tissues. The roles of tumor-associated ESCs on EC cell should be further investigated in future study. In addition, whether normal ESCs or NrCAM administration could reverse progestin insensitivity in EC patients needs more effort.

In this study, we found that ESCs enhanced the inhibitory effect of progestins on EC growth by secreting NrCAM via TET1-induced hydroxymethylation of *PRB* promoter. NrCAM combined with MPA inhibited EC growth to a greater extent than individual treatments, indicating that NrCAM or NrCAM plus progestins could be a new treatment for EC. However, the clinical safety and efficacy of NrCAM need to be comprehensively evaluated in future studies.

## Supplementary information

Supplementary Table

Supplementary Figure

Dataset 1

Dataset 2B1

Dataset 2B2

Dataset 3B1

Dataset 3B2

Dataset 3B3

Dataset 3B4

Dataset 4E1

Dataset 4E2

Dataset 4G1

Dataset 4G2

Dataset 4G3

Dataset 4I1

Dataset 4I2

Dataset 5A1

Dataset 5A2

Dataset 5A3

Dataset 5A4

Dataset 6

Dataset 7

Dataset 8

Dataset 9

## Data Availability

The raw and normalized data gene expression data used in this manuscript have been deposited in the Gene Expression Omnibus repository, with GEO accession number GSE196890.
