# Supplementary material for: NrCAM secreted by endometrial stromal cells enhances the progestin sensitivity of endometrial cancer cells through epigenetic modulation of PRB
Source: Cancer Gene Ther. 2022 Apr 6;29(10):1452–62. doi: 10.1038/s41417-022-00467-0 (PMC9576598; doi:10.1038/s41417-022-00467-0)
Supplement: Supplementary file 1 — Supplementary Table [file 41417_2022_467_MOESM1_ESM.pdf]

Supplementary table 1. General characteristics of the clinical sample

Patient No.	Age (year)	Initial pathological Diagnosis	Treatment	Pathological diagnosis and efficacy during treatment every 3 months						Progestin response
				After 3 months		After 6 months		After 9 months		
				Diagnosis	Treatment result	Diagnosis	Treatment result	Diagnosis	Treatment result	
Case 1#	28	EEC	MA	EAH	PR	secretory reaction	CR	secretory reaction	CR	sensitive
Case 2	29	EEC	MA	secretory reaction	CR	secretory reaction	CR	secretory reaction	CR	sensitive
Case 3	31	EEC	MA	secretory reaction	CR	secretory reaction	CR	/	/	sensitive
Case 4	34	EEC	MA+ LNG-IUS	proliferation rection	CR	proliferation rection	CR	secretory reaction	CR	sensitive
Case 5	32	EEC	MA+ LNG-IUS	EAH	PR	secretory reaction	CR	secretory reaction	CR	sensitive
Case 6	30	EEC	MA	EEC	NR	EAH	PR	EAH	PR	insensitive
Case 7	38	EEC	MA+ LNG-IUS	EAH	PR	EAH	PR	EAH + morular metaplasia	PR	insensitive
Case 8	26	EEC	MA	EAH	PR	CH	PR	EAH	PR	insensitive
Case 9#	20	EEC	MA	EEC	NR	EAH	PR	EEC	NR	insensitive

EAH, endometrial atypical hyperplasia; EEC, endometrioid endometrial carcinoma; CH, complex hyperplasia; PR, partial response; CR, complete response; NR, no response; MA, megestrol acetate; LNG-IUS, levonorgestrel intrauterine system. #, Representative pathological pictures of the two patients during treatment were shown in Figure 1A.

Supplementary table 2. Clinical characteristics of the patients

Case	Age	Pathological Diagnosis	Endometrial cycle	Height (m)	Weight (Kg)	BMI (kg/m^2)	Fertility
1	50	Uterine leiomyoma	Secretory phase	1.65	68	24.977	G2P1
2	48	Uterine leiomyoma	Proliferative phase	1.63	57	21.454	G3P2
3	48	Uterine leiomyoma	Proliferative phase	1.6	73	28.516	G4P2
4	34	Cervical high-grade squamous intraepithelial lesion	Proliferative phase	1.65	60	22.039	G3P1
5	50	Cervical invasive squamous cell carcinoma	Secretory phase	1.6	55	21.484	G4P3
6	48	Uterine leiomyoma	Secretory phase	1.57	53	21.502	G2P1
7	30	Uterine leiomyoma	Proliferative phase	1.72	58.6	19.808	G0P0
8	51	Uterine leiomyoma	Proliferative phase	1.55	52	21.644	G3P1
9	50	Cervical invasive squamous cell carcinoma	Secretory phase	1.7	50	17.301	G2P1
10	45	Cervical invasive squamous cell carcinoma	Proliferative phase	1.6	54	21.094	G4P3
11	45	Uterine leiomyoma	Proliferative phase	1.55	45	18.73	G4P1
12	46	Uterine leiomyoma	Proliferative phase	1.63	77	28.981	G1P1
13	45	Cervical high-grade squamous intraepithelial lesion	Proliferative phase	1.61	72	27.777	G2P1

14	40	Uterine leiomyoma	Proliferative phase	1.65	58.4	21.451	G1P1
15	45	Uterine leiomyoma	Secretory phase	1.56	50	20.546	G2P2
16	41	Cervical invasive squamous cell carcinoma	Secretory phase	1.63	59.3	22.319	G4P1
17	42	Cervical invasive squamous cell carcinoma	Proliferative phase	1.62	51	19.433	G3P1
18	36	Cervical invasive squamous cell carcinoma	Proliferative phase	1.63	66	24.841	G3P1
19	48	Uterine leiomyoma	Secretory phase	1.5	52	23.111	G6P2
20	45	Uterine leiomyoma	Secretory phase	1.65	58	21.304	G2P1
21	47	Cervical high-grade squamous intraepithelial lesion	Proliferative phase	1.58	75.4	30.203	G5P1
22	42	Cervical squamous cell carcinoma in site	Secretory phase	1.59	54.7	21.637	G3P1
23	36	Uterine leiomyoma	Proliferative phase	1.48	46	21.001	G3P2
24	38	Cervical high-grade squamous intraepithelial lesion	Proliferative phase	1.64	58.3	21.676	G1P1
25	29	Uterine leiomyoma	Proliferative phase	1.58	52	20.83	G1P0
26	44	Uterine leiomyoma	Secretory phase	1.62	55	20.957	G3P1
27	45	Cervical invasive squamous cell carcinoma	Proliferative phase	1.61	72	27.777	G2P1
28	45	Uterine leiomyoma	Proliferative phase	1.56	59	24.244	G4P3

29	49	Cervical high-grade squamous intraepithelial lesion	Secretory phase	1.64	65	24.167	G3P1
30	37	Cervical high-grade squamous intraepithelial lesion	Secretory phase	1.6	58	22.656	G1P1

Supplementary table 3. Comparison of basic patient information between Proliferative phase and Secretory phase

	Proliferative phase (n=18)	Secretory phase (n=12)	P value
Median age (years)	45 (29-51)	46.5 (37-50)	P = 0.0632
BMI (Kg/m2)	23.42 ± 3.665	21.83 ± 1.940	P = 0.1811
Uterine leiomyoma	10	6	
Cervical cancer/HSIL	8	6	P > 0.99

Supplementary table 4. Target sequences used for gene silencing

Gene	Sequence
si*PGR*	GCTTCAAGTTAGCCAAGAA
si*NrCAM-1*	GGAGCATGGTGTCCTTTGA
si*NrCAM-2*	GAAGGAGTCTATCAGTGTA
si*NrCAM-3*	GCAGATCATTCATGTTTCA
si*TET1*	GCTCAAACGAGGTCCATTA

Supplementary table 5. Primers used for Real-time PCR

Genes		Primer sequence
GAPDH	Forward	5'-AAGATGTGCCTGTCCTGTGTC-3'
	Reverse	5'-GCTTGTCCTGCTTTCTGTTCTC-3'
PGR	Forward	5'-TCTACCCGCCCTATCTCAACTA-3'
	Reverse	5'-AGAAGACCTTACAGCTCCCACA-3'
TET1	Forward	5'-ACCCCCTGTCACCTGCTGAGG-3
	Reverse	5'-GCGATGGCCACCCCACCAAT-3'
NrCAM	Forward	5'-AGTGTGTGAGTCTCAGCAGG-3'
	Reverse	5'-TGTTGGGTGATGGTTGGAGG-3'
HAND2	Forward	5'-CCTCTTCGTCGGTCTTC-3'
	Reverse	5'-AAGATCAAGACACTGCGCCT-3'
IRF4	Forward	5'-GCGGTGCGCTTTGAACAAG-3'
	Reverse	5'-ACACTTTGTACGGGTCTGAGA-3'
NR2F2	Forward	5'-CGGGTGGTCGCCTTTATGG-3'
	Reverse	5'-ACAGGCATCTGAGGTGAACAG-3'
RGCC	Forward	5'-ACAGACTCTACCCCAGCTCTT-3'
	Reverse	5'-AGAAGGTTGAGTGCACGTCTT-3'
CDH20	Forward	5'-CAGCACTGTCCACAGCTACG-3'
	Reverse	5'-TATTGCTCGTGTGGTTGGCT-3'
MTIG	Forward	5'-CTTCTCGCTTGGGAACTCTA-3'
	Reverse	5'-AGGGGTCAAGATTGTAGCAAA-3'
SLC28A1	Forward	5'-CGCCGCAGGATTGCCTTAT-3'
	Reverse	5'-CCAGTGAAATTGCCCTTGCC-3'
HSD11B1	Forward	5'-CGAAATCTTGAGGTTCTCTCTG-3'
	Reverse	5'-AAGCTCCAGGCAGTGGGATA-3'
ANGPTL4	Forward	5'-GCTTCGCCTTCTTGCTGA-3'
	Reverse	5'-AACAGCGCGCTCGAGAAG-3'
TRABD2B	Forward	5'-GGAGATTGACAGCTACTTCCG-3'
	Reverse	5'-CAGAAAGTGACCTGCTCCGAA-3'
ERVMER34-1	Forward	5'-AGCGGGAGAAAATCACCCAG-3'

	Reverse	5'-AGTCGCGGTTGTCCAGACT-3'
WISP1	Forward	5'-CTACAACAACGGCCAGTCCT-3'
	Reverse	5'-ACATACCCACTGCTCACAGC-3'
CPM	Forward	5'-GCGCTGGATTTCAACTACCAC-3'
	Reverse	5'-TCCCGCCCAACAGTCTCAT-3'
ITGA10	Forward	5'-AACATCACCCACGCCTATTCC-3'
	Reverse	5'-GTTGGTAGTCACCTAAGTGGC-3'

Supplementary table 6. *PRB* promoter fragment primers used for Hmedip

Gene		Primer sequence
PGR1	Forward	5'-CAGCCAGAGCCCACAATACA -3'
	Reverse	5'- GTTGTGCTGCCCTTCCATTG -3'
PGR2	Forward	5'- CTTCCCTCGGCCCTGCCAGGG -3'
	Reverse	5'- AGGGCGCCAACAGAGTGTCC -3'
PGR3	Forward	5'- ACCCCCTGTCACCTGCTGAGG -3'
	Reverse	5'- GGCTTACCCCGATTAGTGAC-3'
