# Supplementary material for: NrCAM secreted by endometrial stromal cells enhances the progestin sensitivity of endometrial cancer cells through epigenetic modulation of PRB
Source: Cancer Gene Ther. 2022 Apr 6;29(10):1452–62. doi: 10.1038/s41417-022-00467-0 (PMC9576598; doi:10.1038/s41417-022-00467-0)
Supplement: Supplementary file 2 — Supplementary Figure [file 41417_2022_467_MOESM2_ESM.pdf]

Supplementary Figure 1. Identification of ESCs and culture of ESCs and HLF-1

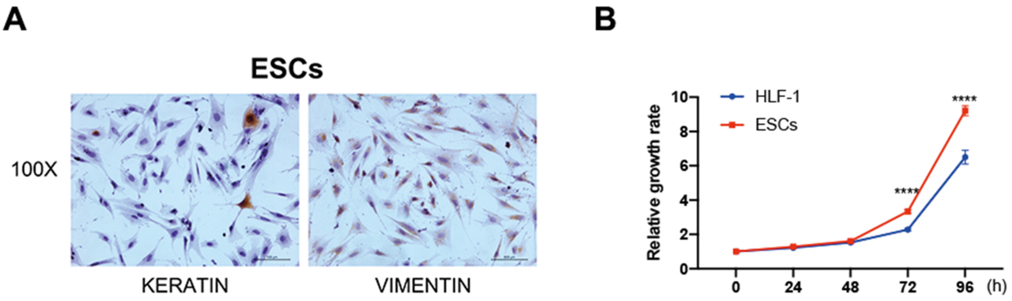

A. Vimentin-positive and Keratin-negative expression was used to identify ESCs by ICC staining. Original magnification, 200×; Scale bar, 100μm. B. Comparison of proliferation of ESCs and HLF-1. ESCs and HLF-1 were cultured in DMEM/F12 for 24, 48, 72, and 96 h before CCK-8 assays. ****, P<0.0001.

Supplementary Figure 2. ESCs had consistent inhibitory effects on EC cells

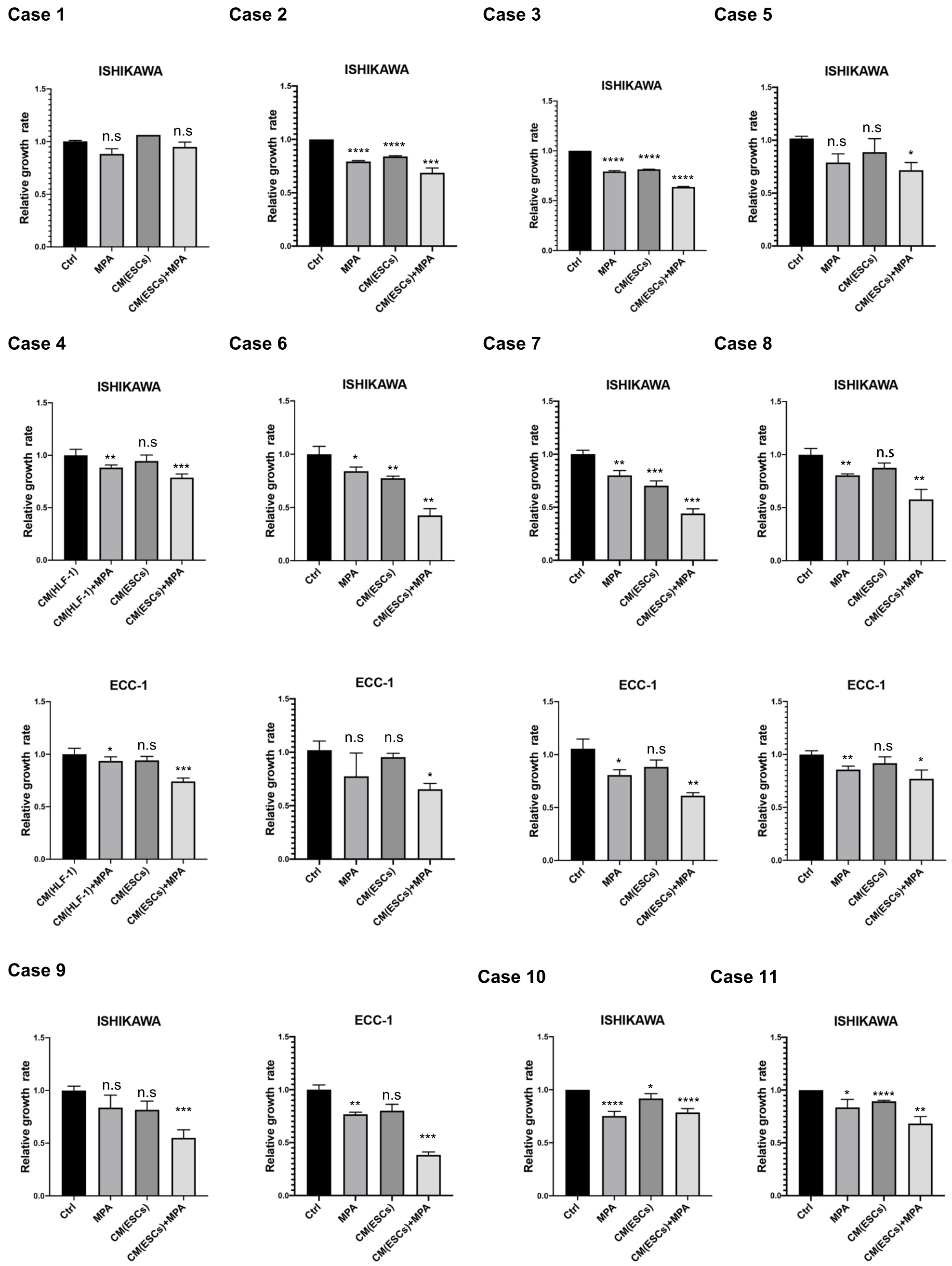

ESCs derived from case 1 to case 11 (7 cases in proliferative phase and 4 cases in secretory phase) had consistent inhibitory effects on EC cells by CCK-8 assays. *, P<0.05; **, P<0.01; ***, P<0.001; ****, P<0.0001; n.s, not significant.

Supplementary Figure 3. The effect of BMP2, ITGA10, and WISP1 on EC cell growth with or without MPA treatment

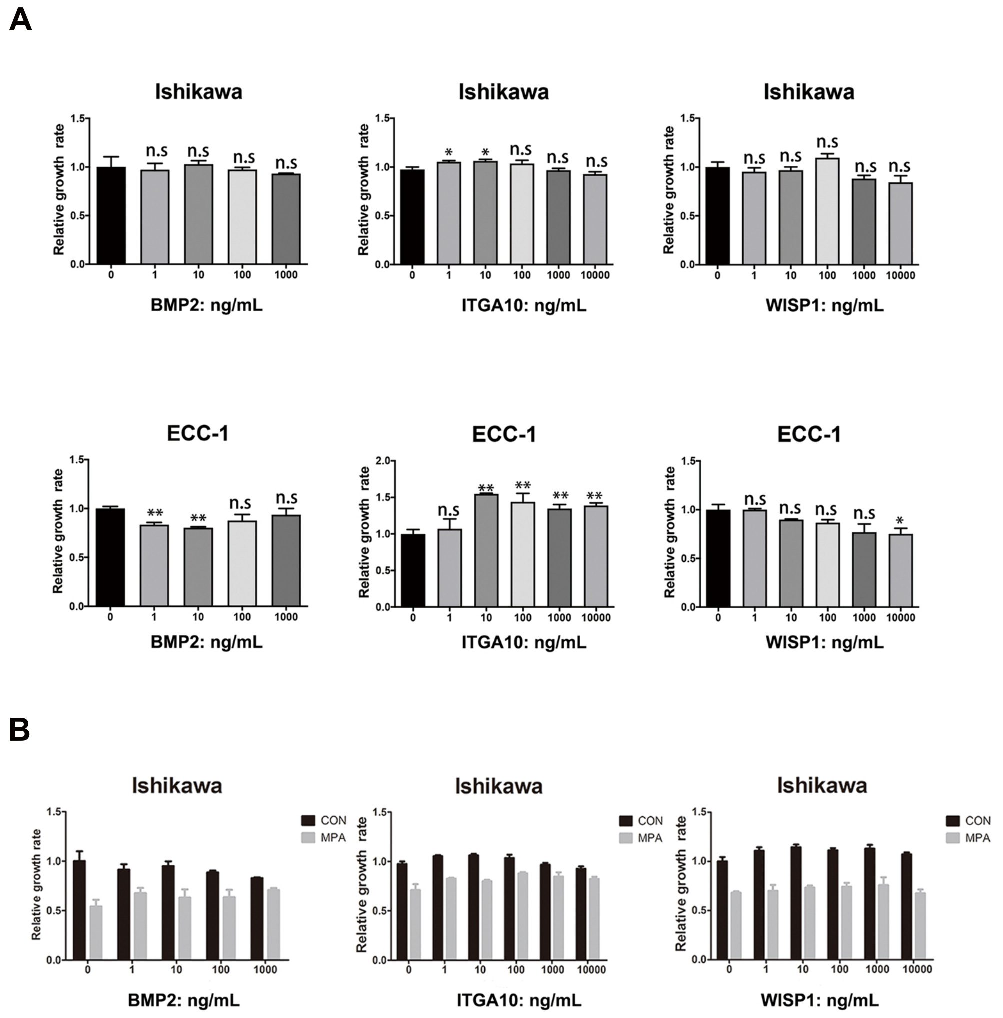

A. The effect of BMP2, ITGA10, and WISP1 on EC cell proliferation. Ishikawa and ECC-1 cells were treated with the indicated doses of BMP2, ITGA10, and WISP1 for 48 h before CCK-8 assays.

B. The effect of BMP2, ITGA10, and WISP1 with or without MPA on the proliferation of EC cells. Ishikawa cells were treated with the indicated doses of BMP2, ITGA10, and WISP1 and/or 10 μM MPA for 48 h before CCK-8 assays. *, $P<0.05$; **, $P<0.01$; n.s, not significant.
