# Supplementary figures and images for: NrCAM secreted by endometrial stromal cells enhances the progestin sensitivity of endometrial cancer cells through epigenetic modulation of PRB
Source: Cancer Gene Ther. 2022 Apr 6;29(10):1452–62. doi: 10.1038/s41417-022-00467-0 (PMC9576598; doi:10.1038/s41417-022-00467-0)

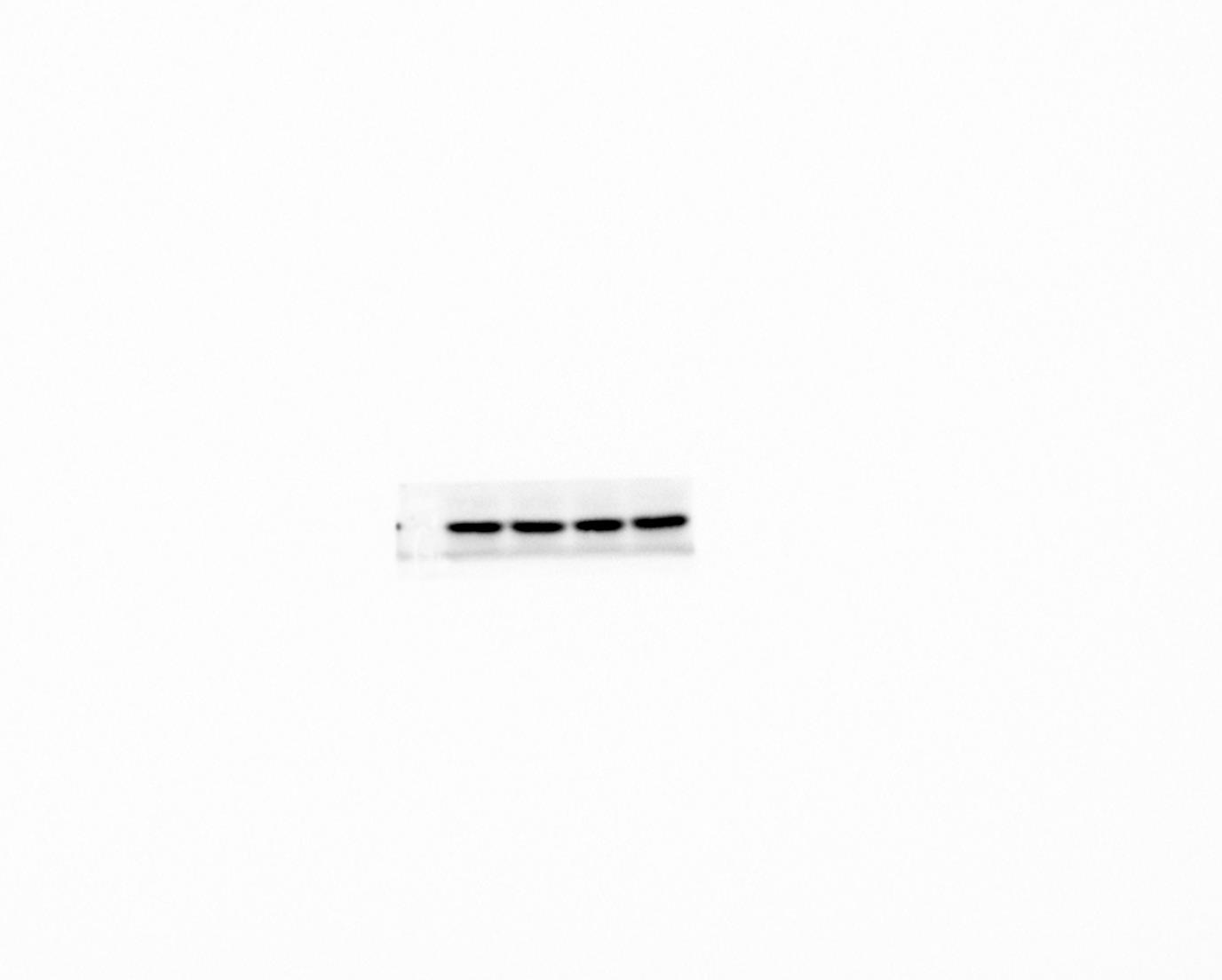

Supplement: Supplementary file 4 — Dataset 2B1 [file 41417_2022_467_MOESM4_ESM.jpg]

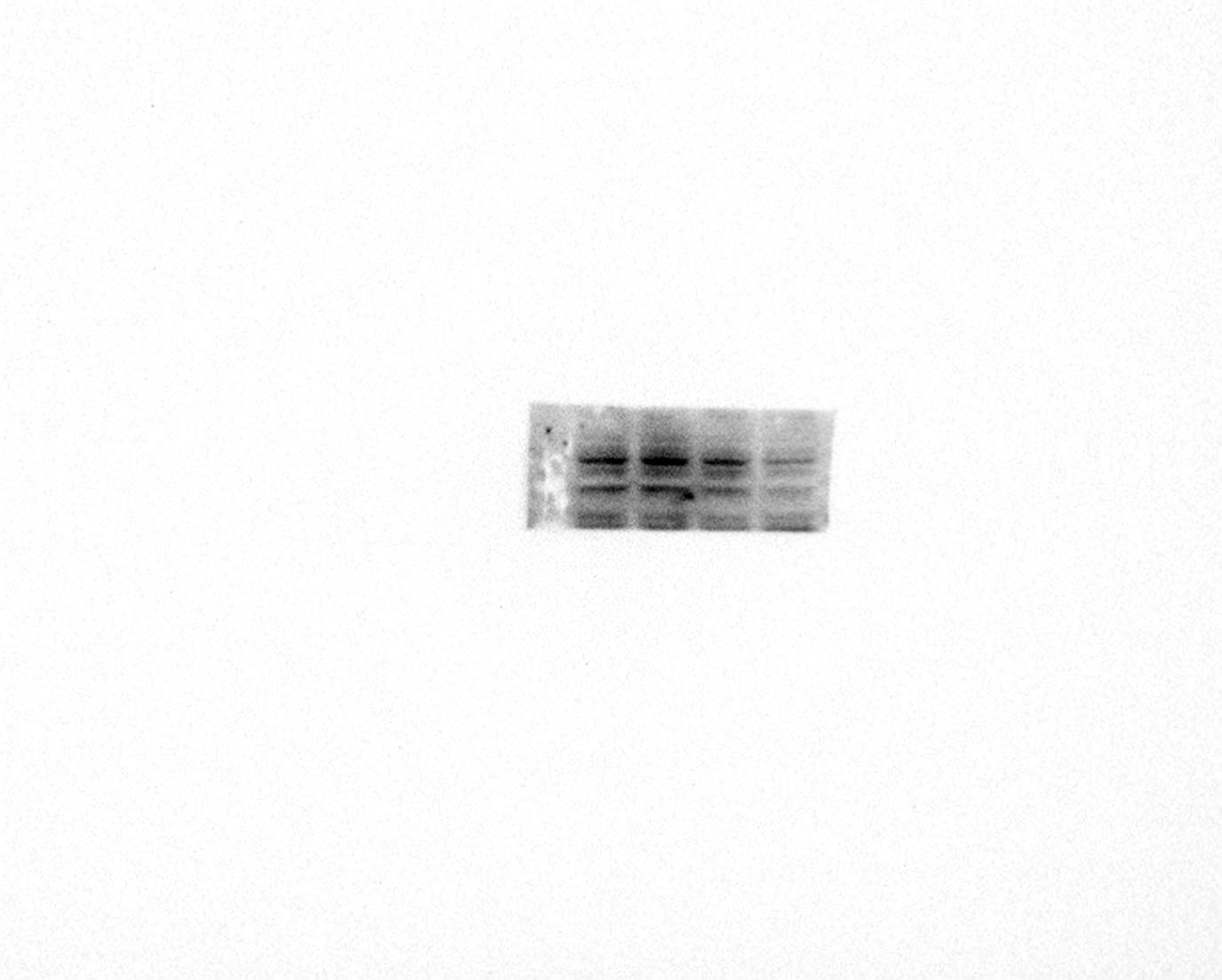

Supplement: Supplementary file 5 — Dataset 2B2 [file 41417_2022_467_MOESM5_ESM.jpg]

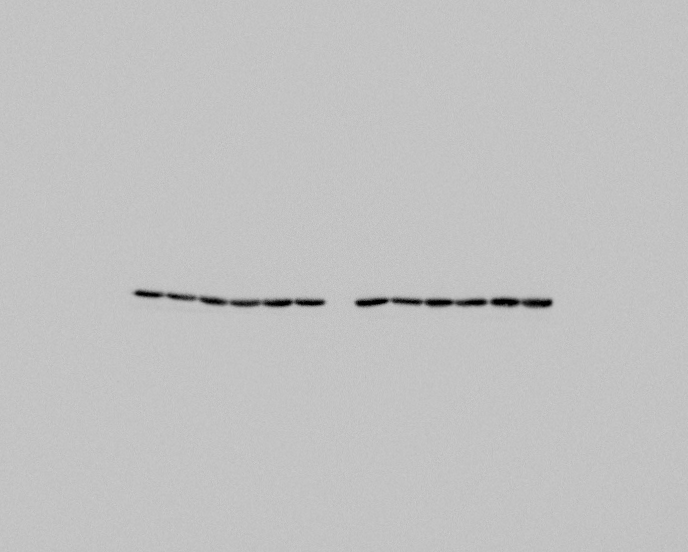

Supplement: Supplementary file 6 — Dataset 3B1 [file 41417_2022_467_MOESM6_ESM.tif]

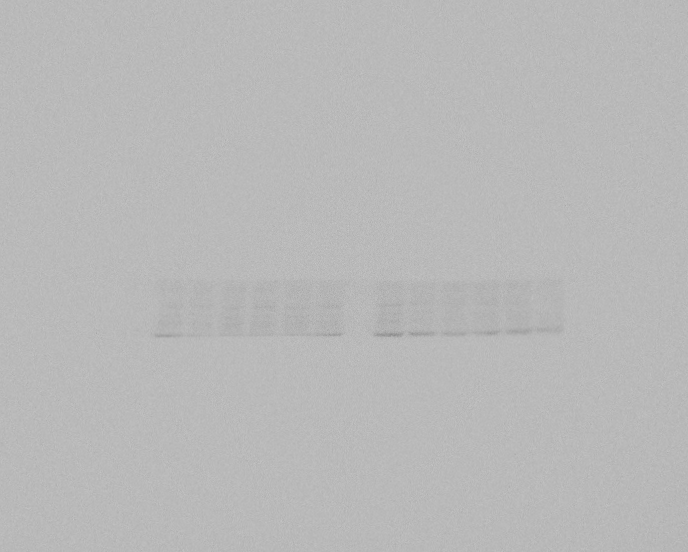

Supplement: Supplementary file 7 — Dataset 3B2 [file 41417_2022_467_MOESM7_ESM.tif]

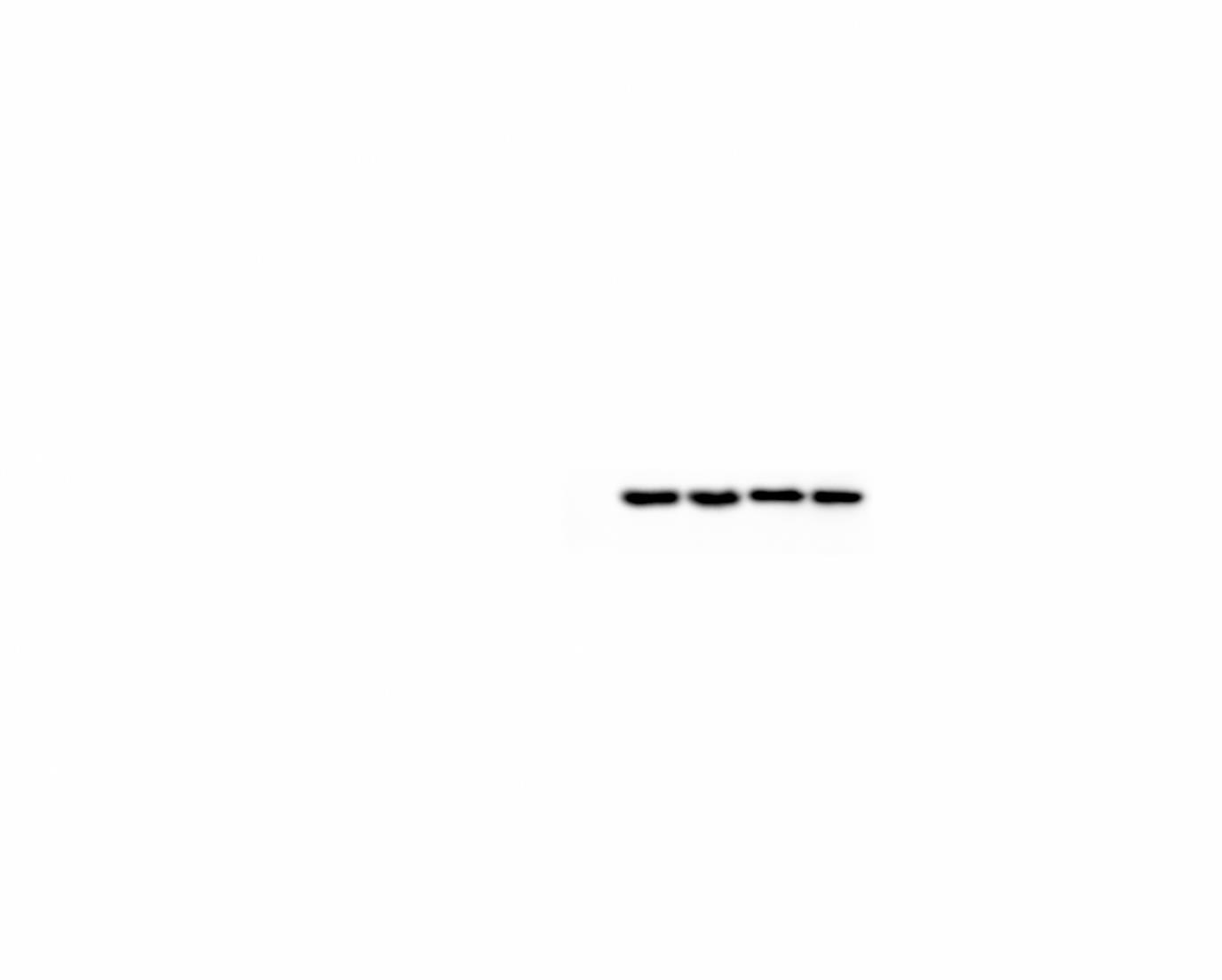

Supplement: Supplementary file 10 — Dataset 4E1 [file 41417_2022_467_MOESM10_ESM.jpg]

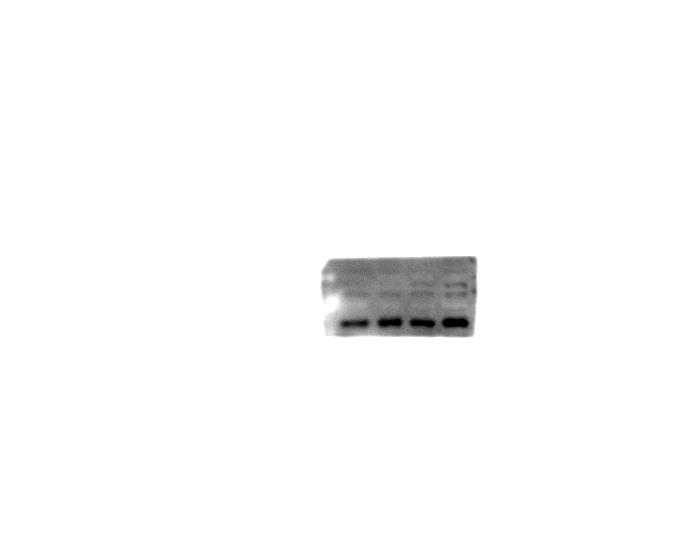

Supplement: Supplementary file 11 — Dataset 4E2 [file 41417_2022_467_MOESM11_ESM.jpg]

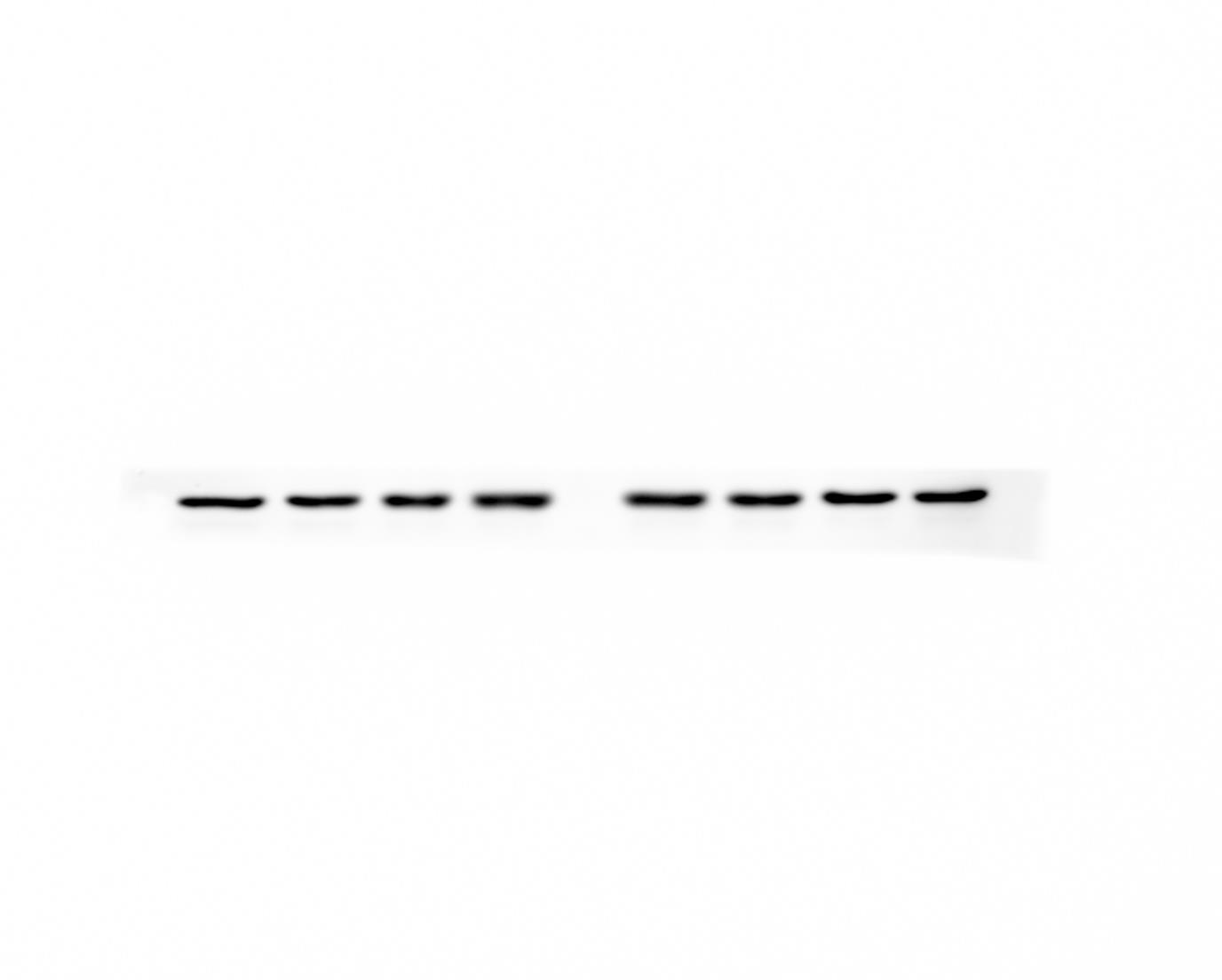

Supplement: Supplementary file 12 — Dataset 4G1 [file 41417_2022_467_MOESM12_ESM.jpg]

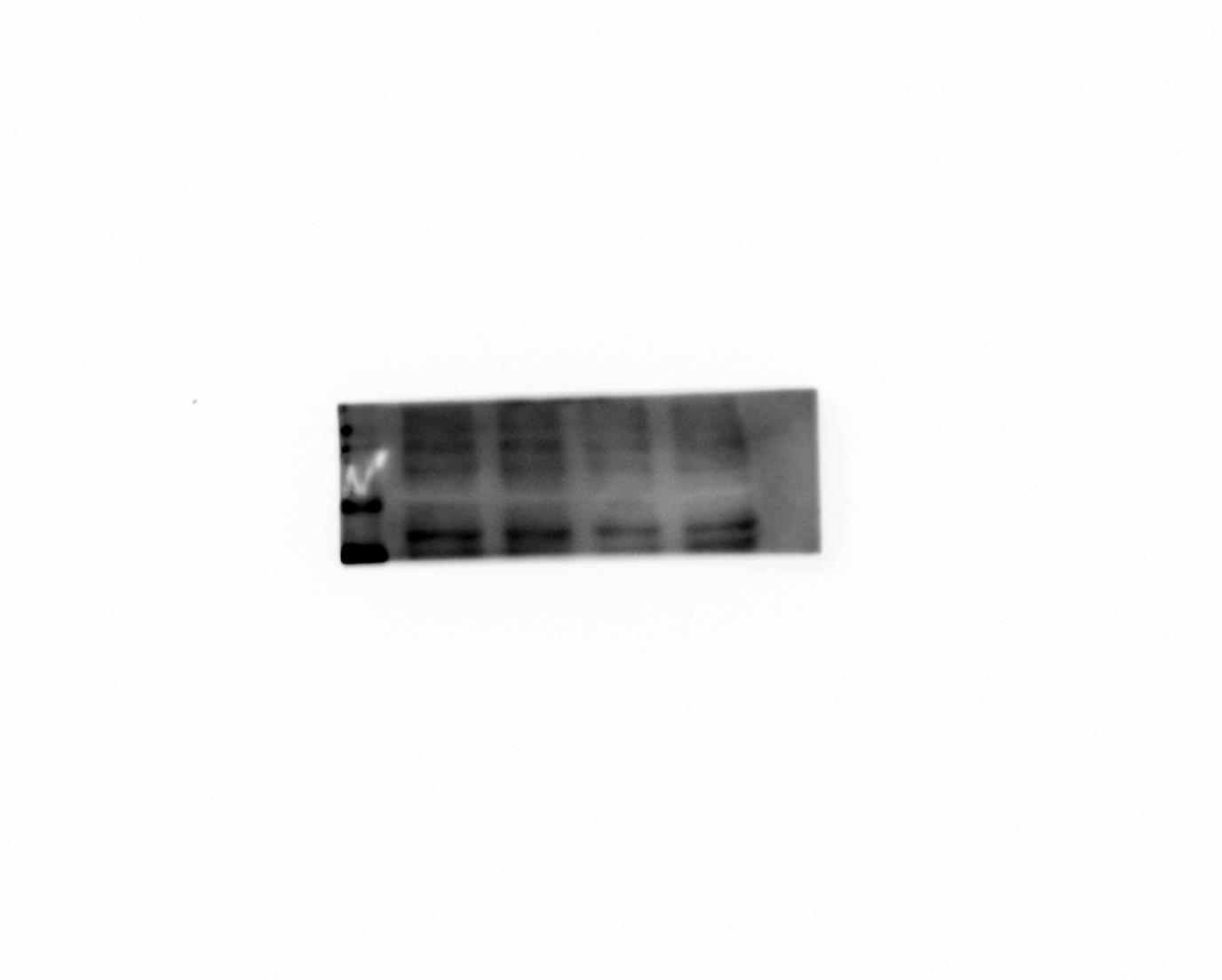

Supplement: Supplementary file 13 — Dataset 4G2 [file 41417_2022_467_MOESM13_ESM.jpg]

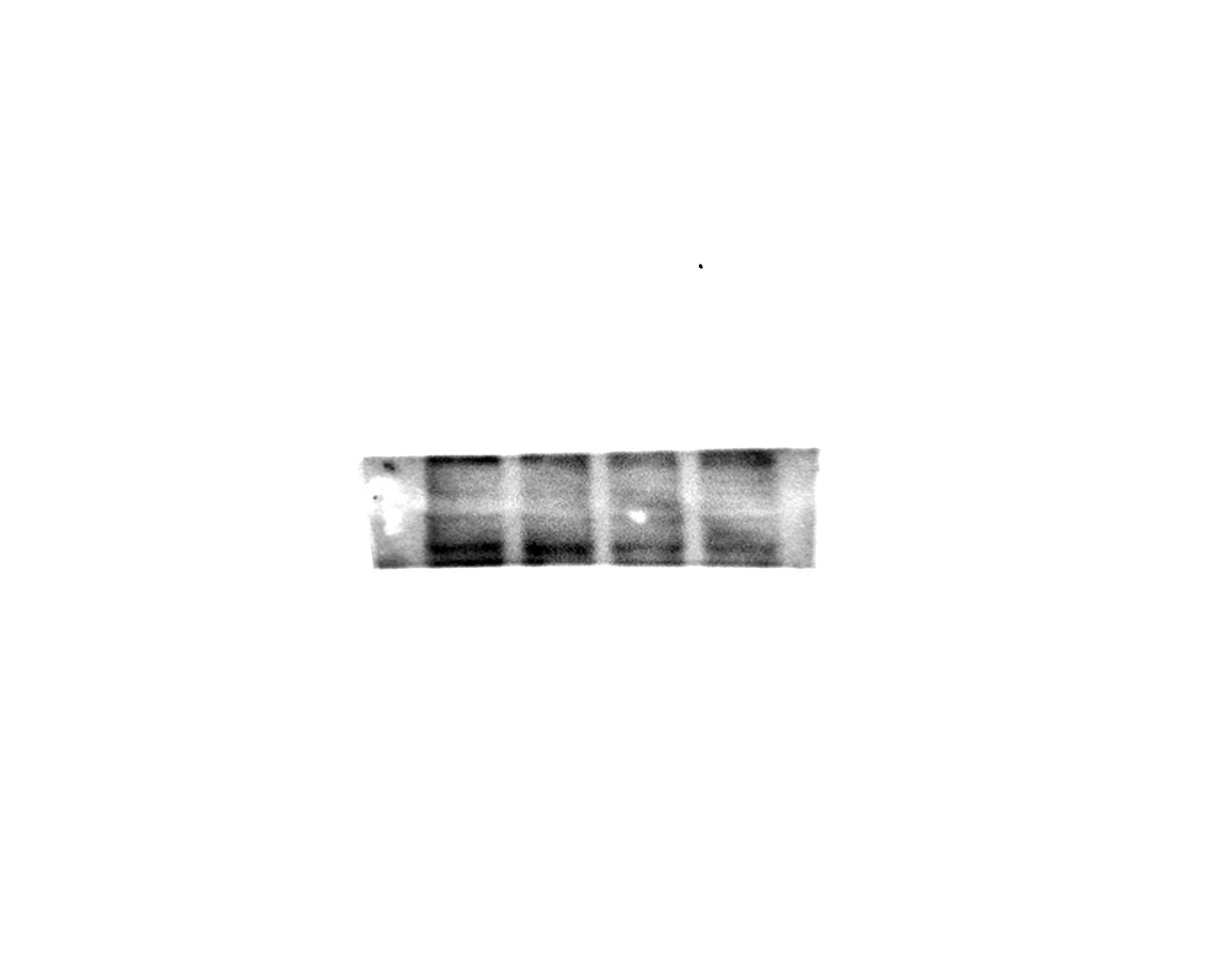

Supplement: Supplementary file 14 — Dataset 4G3 [file 41417_2022_467_MOESM14_ESM.jpg]

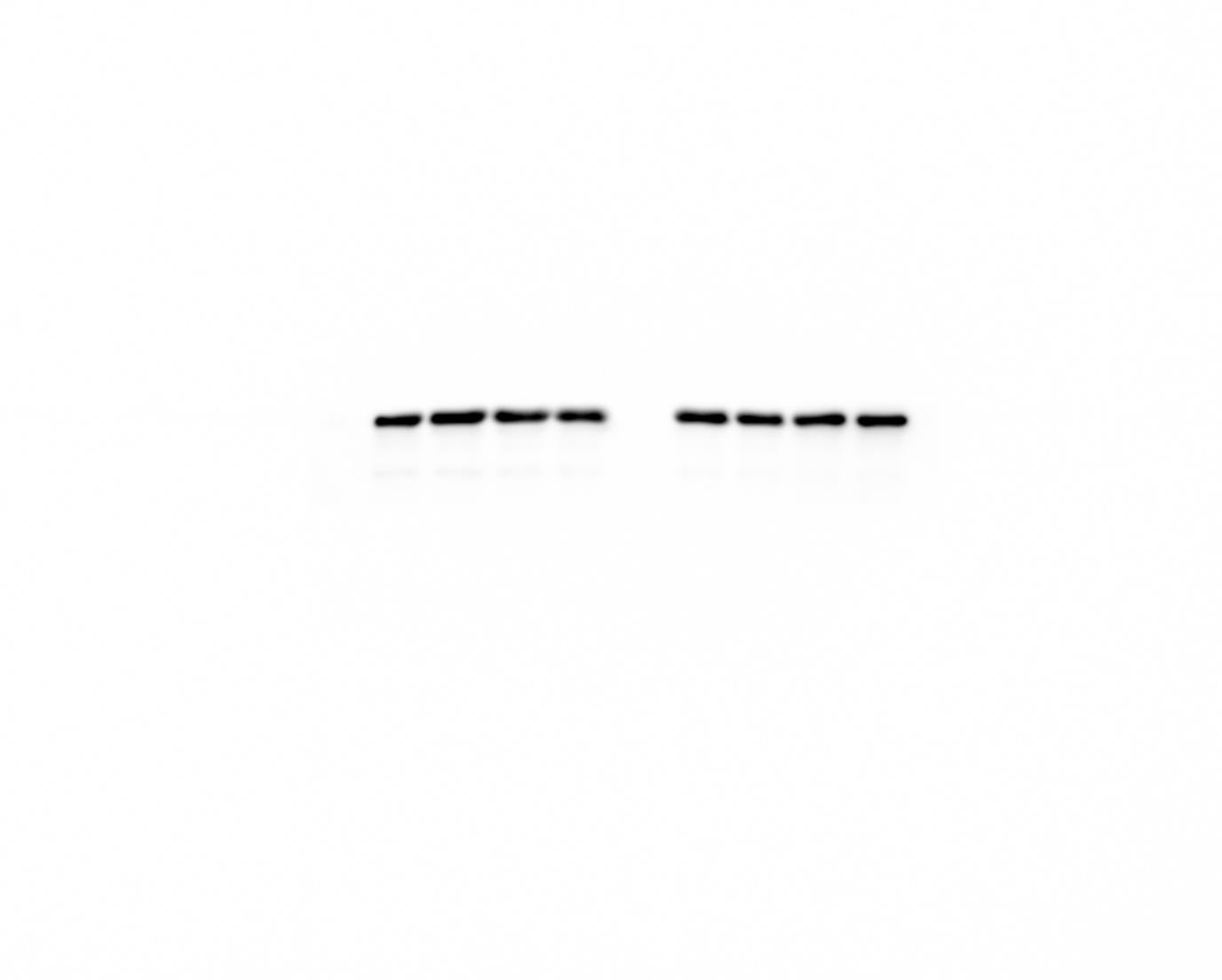

Supplement: Supplementary file 15 — Dataset 4I1 [file 41417_2022_467_MOESM15_ESM.jpg]

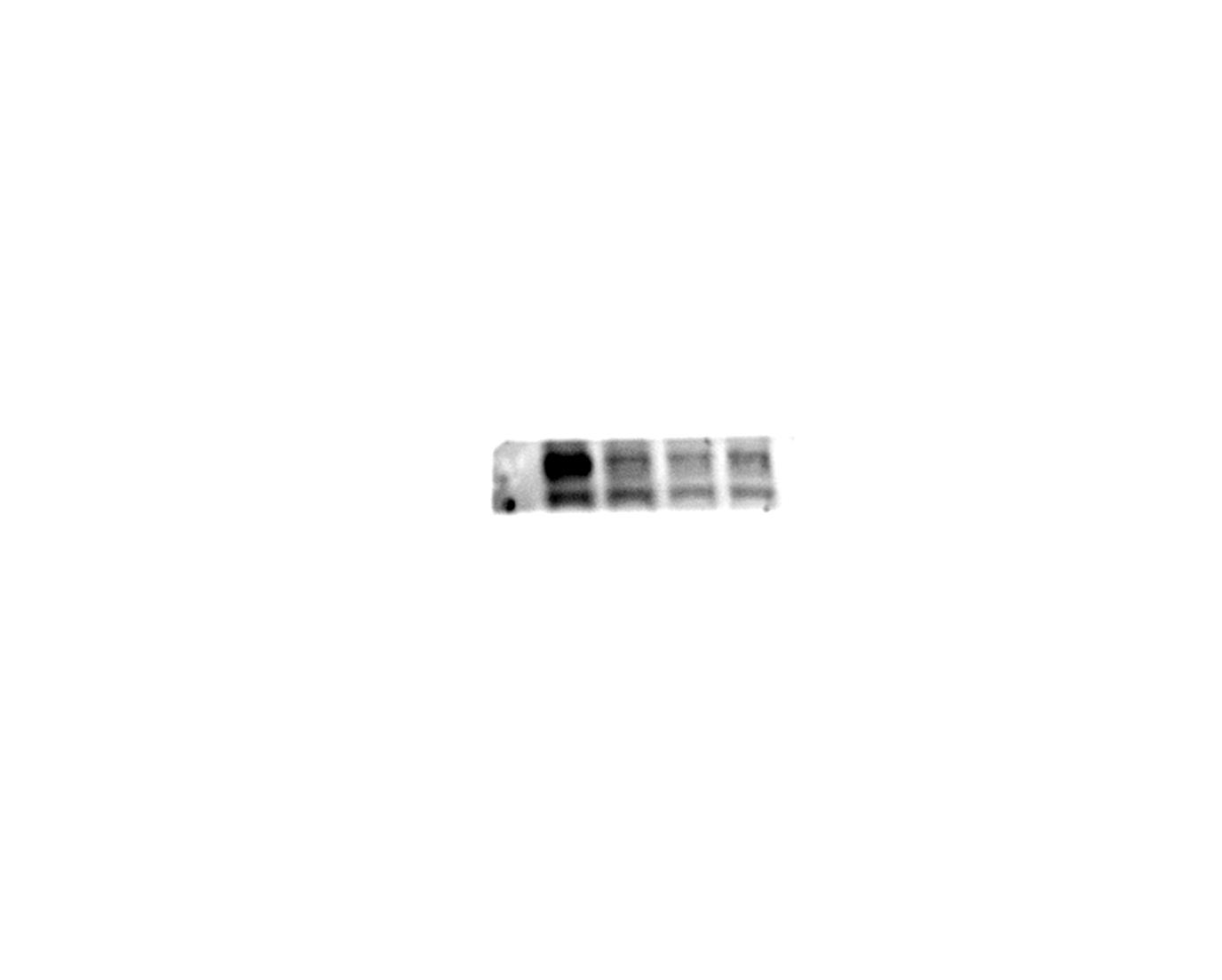

Supplement: Supplementary file 16 — Dataset 4I2 [file 41417_2022_467_MOESM16_ESM.jpg]

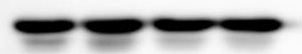

Supplement: Supplementary file 17 — Dataset 5A1 [file 41417_2022_467_MOESM17_ESM.jpg]

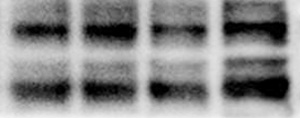

Supplement: Supplementary file 18 — Dataset 5A2 [file 41417_2022_467_MOESM18_ESM.jpg]

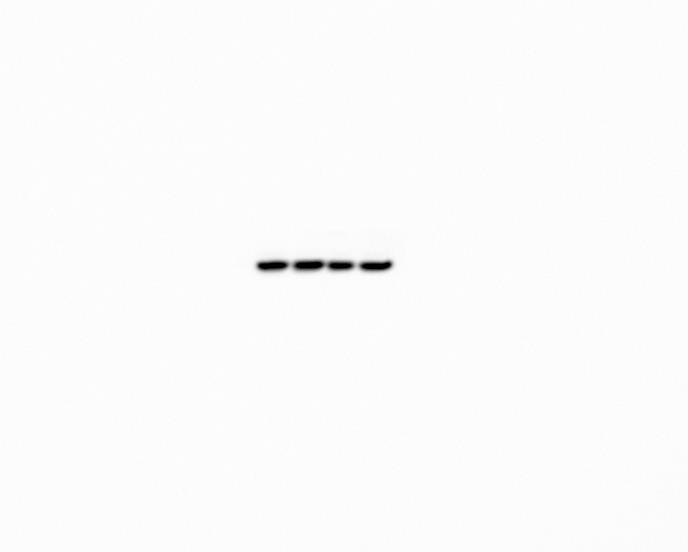

Supplement: Supplementary file 19 — Dataset 5A3 [file 41417_2022_467_MOESM19_ESM.jpg]

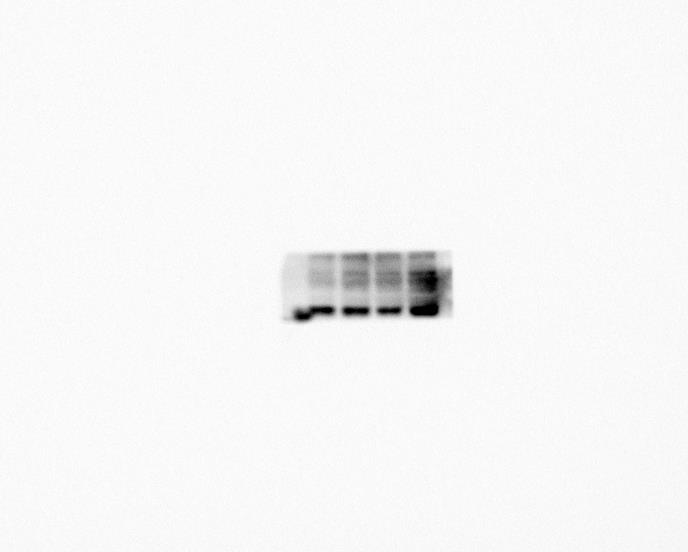

Supplement: Supplementary file 20 — Dataset 5A4 [file 41417_2022_467_MOESM20_ESM.jpg]
